# Protecting maize from rootworm damage with the combined application of arbuscular mycorrhizal fungi, *Pseudomonas* bacteria and entomopathogenic nematodes

**DOI:** 10.1038/s41598-019-39753-7

**Published:** 2019-02-28

**Authors:** Geoffrey Jaffuel, Nicola Imperiali, Kent Shelby, Raquel Campos-Herrera, Ryan Geisert, Monika Maurhofer, Joyce Loper, Christoph Keel, Ted C. J. Turlings, Bruce E. Hibbard

**Affiliations:** 1 FARCE Laboratory, Institute of Biology, University of Neuchâtel, Neuchâtel, Switzerland; 2 Department of Fundamental Microbiology, University of Lausanne, Lausanne, Switzerland; 3 Biological Control of Insects Research, US Department of Agriculture, Agricultural Research Service, Columbia, MO USA; 4 Instituto de Ciencias de la Vid y del Vino, CSIC-Universidad de La Rioja-Gobierno de La Rioja, Logroño, Spain; 5 Institute of Integrative Biology, ETH Zurich, Zurich, Switzerland; 6 Department of Botany and Plant Pathology, Oregon State University, Corvallis, OR USA; 7 Horticultural Crops Research Laboratory, US Department of Agriculture, Agricultural Research Service, Corvallis, OR USA; 8 Plant Genetics Research Unit, US Department of Agriculture-ARS, University of Missouri, Columbia, MO USA

## Abstract

*Diabrotica virgifera virgifera* LeConte, the western corn rootworm (WCR), is the most destructive pest of maize in North America, and has recently spread across central Europe. Its subterranean larval stages are hard to reach with pesticides and it has evolved resistance to conventional management practices. The application of beneficial soil organisms is being considered as a sustainable and environmental friendly alternative. In a previous study, the combined application in wheat fields of arbuscular mycorrhizal fungi, entomopathogenic *Pseudomonas* bacteria, and entomopathogenic nematodes was found to promote growth and protection against a natural pest infestation, without negative cross effects. Because of the insect-killing capacity of the bacteria and nematodes, we hypothesized that the application of these organisms would have similar or even greater beneficial effects in WCR-infested maize fields. During three consecutive years (2015–2017), we conducted trials in Missouri (USA) in which we applied the three organisms, alone or in combinations, in plots that were artificially infested with WCR and in non-infested control plots. For two of the three trials, we found that in plots treated with entomopathogenic nematodes and/or entomopathogenic *Pseudomonas* bacteria, roots were less damaged than the roots of plants in control plots. During one year, WCR survival was significantly lower in plots treated with *Pseudomonas* than in control plots, and the surviving larvae that were recovered from these plots were lighter. The bacterial and nematodes treatments also enhanced yield, assessed as total grain weight, in one of the trials. The effects of the treatments varied considerable among the three years, but they were always positive for the plants.

## Introduction

*Diabrotica virgifera virgifera* LeConte, the western corn rootworm (WCR), causes significant damage to maize (*Zea mays* L.) across North America, as well as across Central and Eastern Europe^1,2^. The larval stage is the most damaging, as it feeds on root hairs, cortical tissue, and tunnels inside the roots of maize plants. This can lead to the destruction of roots^3,4^, which hampers the uptake of water and nutrients from the soil^5^, and increases plant’s susceptibility to lodging^6^. Often, roots are fully pruned by older larvae that move up to the base of the stalk^7^. In affected areas in the US, WCR larvae can cause tremendous yield losses^1,8–10^.

From the time that it was discovered as a pest^11^ until 1946, the only successful management option was crop rotation. Since then, WCR management has also included granular and liquid soil insecticides, and more recently insecticidal seed treatments and transgenic Bt maize^12–14^. Over time, WCR has developed resistance to most insecticides classes^15–17^. Crop rotation is still highly effective against the WCR in most regions, but some populations have apparently lost their ovipositional fidelity to cornfields, and lay eggs in soybean and other crops in addition to maize^18–20^. Beginning in 2003, transgenic maize carrying a gene from the entomopathogenic bacterium *Bacillus thuringiensis* Berliner (Bt) has been effective in controlling the WCR and northern corn rootworms (*D. barberi*). Yet, certain WCR populations have since evolved resistance to some Bt toxins^21^. This ability of WCR to rapidly evolve resistance has significantly reduced the efficacy of these management strategies, at least in certain areas.

Kuhlmann & Van der Burgt^22^ recommended biological control as an option for Europe, where genetically modified plants are mostly banned and the use of additional insecticides is not desirable. Classical biological control would involve the importation and the establishment of natural enemies from the WCR area of origin in North America. A more readily available option would be an inundative biological control approach with commercially available native antagonists, such as entomopathogenic nematodes (EPN)^22^.

Soil-dwelling EPN have been successfully used as biological control agents against a range of different insect pests, including WCR^23–25^. EPN are favored because they are harmless to vertebrates, commercially available, and authorized in many countries^26–31^. EPN in the families Steinernematidae and Heterorhabditidae carry mutualistic bacteria of the genera *Xenorhabdus* and *Photorhabdus*, respectively, and together function as obligate parasites of insects^32,33^. The free-living stage of EPN, known as the infective juvenile (IJ), is adapted to persist in the soil where it searches for a suitable insect host^34^. Upon contact with a host, it enters the insect’s hemocoel through natural openings and releases their symbiont bacteria. Within 2–3 days, the insect host dies of septicemia caused by the proliferating bacteria. The EPN consume the bacteria and reproduce to form two to three generations, until the resources in the cadaver are depleted. Non-feeding infective stages then emerge and may survive in the surrounding soil for several months in search of a new host^35^.

Various other soil organisms also have the potential to improve plant performance by, for instance, promoting growth, facilitating nutrient acquisition, stimulating defenses, and protecting plants from pathogens and pests^36–38^. Among these are arbuscular mycorrhizal fungi, which colonize roots of many terrestrial plants and can provide these plants with nutrients in exchange for photosynthetic by-products^39,40^. Arbuscular mycorrhizal fungi have also been shown to increase plant tolerance to a variety of stresses, both biotic and abiotic^40^. Some arbuscular mycorrhizal fungi such as *Rhizoglomus irregularis* are commercialized as inoculates for seedlings or as seed coatings, in order to improve soil fertility and plant performance^41–46^.

Similarly, growth promoting rhizobacteria within the *Pseudomonas fluorescens* group, such as *Pseudomonas protegens* and *Pseudomonas chlororaphis*, have been shown to trigger systemic resistance in colonized plants, and may control soil-borne pathogens with potent antifungal compounds^47–51^. *Pseudomonas protegens* and *Pseudomonas chlororaphis* strains also have insecticidal activity and are particularly effective against Lepidopteran pests^52–55^. Currently there are several products based on plant-beneficial pseudomonads that are commercialized, primarily in the USA^52,56–58^.

A previous study^59^ showed that the combined application of the EPN *Heterorhabditis bacteriophora* and the rhizobacteria *Pseudomonas protegens* CHA0 and *Pseudomonas chlororaphis* PCL1391 improved the performance and protection of wheat. This was most evident during a season that the plants were infested by frit fly larvae^59^.

In the current study, we evaluated the singular application of three beneficial soil organisms on maize performance under WCR infestation. Treatments with EPN (*Steinernema feltiae* and *H. bacteriophora*), *Pseudomonas* bacteria, and a commercial formulation of arbuscular mycorrhizal fungi, as well as a treatment with the combination of all three beneficial organisms were applied under realistic field conditions.

## Materials and Methods

### The beneficial soil organisms’ origins and formulations

Strains of *Pseudomonas protegens* Pf-5^60,61^ and *Pseudomonas chlororaphis* O6^62^ with a spontaneous resistance to the antibiotic rifampicin were used in this study in 2015 (Table 1). In 2016 and 2017 we used two closely related bacterial strains, *Pseudomonas protegens* CHA0^63^, and *Pseudomonas chlororaphis* PCL1391^64^ that have been similarly selected for spontaneous resistance to rifampicin following previously described protocols^59,65^ (Table 1). To prepare the bacterial inoculum for field application, the strains were grown overnight at 25 °C in LB Broth Miller (Fisher BioReagents) containing 100 µg/ml of rifampicin. Aliquots of 200 µl of each culture were then plated on LB Agar Miller (Fisher BioReagents) without antibiotics. After incubation at 27 °C for 16 h, bacterial cells were harvested and washed in sterile distilled water. The optical density at 600 nm (OD_600_) of the bacterial cell suspensions was adjusted to 0.15 corresponding to a cell density of about 8 × 10^7^ CFU/ml. To preserve the bacterial concentrations chosen for application to the field, the bacterial stock suspensions were maintained on ice until final dilution and use.

**Table 1:** Beneficial soil organisms applied individually or in combinations in the field experiments.

Beneficial group/species	Strain	Application type	GenBank accession no.	Reference or source
**Arbuscular mycorrhizal fungi**				
*Rhizophagus irregularis* ^a^		Substrate	n.a.^d^	Mycorrhizal Fungi Products Sarasota, Florida
*Funneliformis mosseae* ^a^		Substrate	n.a.	Mycorrhizal Fungi Products Sarasota, Florida
*Septoglomus desertícola* ^a^		Substrate	n.a.	Mycorrhizal Fungi Products Sarasota, Florida
*Claroideoglomus claroideum* ^a^		Substrate	n.a	Mycorrhizal Fungi Products Sarasota, Florida
*Claroideoglomus etunicatum* ^a^		Substrate	n.a	Mycorrhizal Fungi Products Sarasota, Florida
*Rhizoglomus microaggregatum* ^a^		Substrate	n.a	Mycorrhizal Fungi Products Sarasota, Florida
*Rhizoglomus clarum* ^a^		Substrate	n.a	Mycorrhizal Fungi Products Sarasota, Florida
**Entomopathogenic nematodes**				
*Heterorhabditis bacteriophora* ^b^		Aqueous	KJ938576	Koppert biological systems
*Steinernema feltiae* ^b^		Aqueous	KJ938569	Koppert biological systems
***Pseudomonas*** **bacteria**				
*Pseudomonas chlororaphis*	PCL1391^c^	Aqueous	NZ_LFUT01000004	Chin-A-Woeng *et al*.^64^; Flury *et al*.^54^
*Pseudomonas protegens*	CHA0^c^	Aqueous	NC_021237	Stutz *et al*.^63^; Flury *et al*.^54^
*Pseudomonas chlororaphis*	O6^c^	Aqueous	NZ_CM001490.1	Loper, *et al*.^93^
*Pseudomonas protegens*	Pf-5^c^	Aqueous	NC_004129.6	Loper, *et al*.^93^

^a^A commercialized treatment (Ecovam™ Vamendo Granular) containing seven species of arbuscular mycorrhiza was used as inoculant in the 2015, 2016 and 2017 field trials.

^b^A mixture of the entomopathogenic nematodes *H. bacteriophora* and *S. feltiae* was used in the 2015, 2016 and 2017 field trials.

^c^Rifampicin-resistant variants of strains O6 and Pf-5 were used as inoculants in the 2015 field trial, while strains CHA0 and PCL1391 were used as inoculants in the 2016 and 2017 field trials.

^d^n.a., not available.

Entomopathogenic nematodes (EPN) of the species *Steinernema feltiae* and *Heterorhabditis bacteriophora* were provided by the company Koppert Biological Systems (https://www.koppert.com, Table 1). EPN were received in vermiculite powders around two weeks before their application to the field. One or two days before field application, IJs concentration was assessed and the powder containing nematodes weighted to reach a concentration of 0.65 Mio of IJs of each species and placed in a 50 ml sterile conical tube (USA Scientific) (Table 1). Tubes containing the IJs were kept at ~5 °C prior to field application.

Arbuscular mycorrhizal fungi (AMF) were provided by Evocam™ (https://horticulturalalliance.com/product/ecovam-vam-endo-granular/) that contains seven species of arbuscular mycorrhizal fungi, belonging to the genera *Rhizophagus*, *Funneliformis*, *Septoglomus*, *Claroideoglomus* and *Rhizoglomus* (Table 1). The product richness was estimated to 150 spores per gram of substrate. Moreover, a “mock” inoculum, which consisted of the substrate without arbuscular mycorrhizal fungi spores was prepared by autoclaving the original arbuscular mycorrhizal fungi inoculum for 2 h at 110–120 °C, two weeks before field application. Bags containing the inoculum and the “mock” inoculum were stored at room temperature prior to field application.

### Field experiments

Field experiments were conducted during three consecutive springs, in 2015, 2016 and 2017, at the Bradford Research and Extension Centre (38.8929376 N, −92.2009539 W, Columbia, MO, USA). The soil type at this location is a Mexico silt loam made up of 12.5% sand, 65% silt, and 22.5% clay as determined by the University of Missouri Soil Testing Facility, Columbia, MO.

In plots of 1.5 m we planted a row with 8 seeds of the maize cultivar Pioneer 33T55. Each of these experimental rows was separated with a buffer row of the same size planted with the same maize cultivar. Row spacing was 0.76 m, hence, rows were separated from each other by 0.76 m. Experimental plots were hand planted in May of each year. The treatments applied to the field were: (1) EPN suspension, (2) plant-growth promoting rhizobacteria (PGPR) suspension, (3) AMF inoculum, (4) a combination of the EPN, AMF and PGPR, (5) AMF “mock” inoculum, and (6) control (no application). Each year, the experiment was conducted in different fields on the same experimental farm.

Bacterial cell suspensions were applied directly on the maize seeds after they were placed in the furrows using treatment-specific watering cans. Concentrated bacterial stock suspensions (OD_600_ 0.15; corresponding to ~ 8 × 10^7^ CFU/ml) were diluted in ca. 5 L of water for each plot directly at the field site before soil inoculation. In the field trial performed in 2015, the bacterial inoculum was a mixture of *P. protegens* Pf-5 and *P. chlororaphis* O6, while in 2016 and 2017 the chosen strains were *P. protegens* CHA0 and *P. chlororaphis* PCL1391.

For EPN application (*S. feltiae* and *H. bacteriophora*) the nematodes that had been stored in 50 ml sterile tubes were mixed in treatment-specific watering cans in which water was added to a final volume of ca. 5 L per plot and applied in the furrows at a final concentration of 1.3 × 10^6^ IJs/m^2^.

Finally, 400 ml of substrate per plot, containing approximately 4.8 × 10^7^ AMF spores were evenly applied on the seeds using a 500 ml glass beaker. AMF-control plots were inoculated with the same amount of substrate without AMF propagules. Control plots were treated with the same volume of water without the beneficial organisms. After treatments, the seeds were immediately covered with soil by closing the seed furrows. All material which entered into contact with the different inoculants was cleaned and disinfested with 70% ethanol.

When plants were at the two-leaf stage, half of experimental plots were artificially infested with WCR eggs as previously described in El Khishen *et al*.^66^. The WCR eggs were obtained from the USDA-ARS facility in Brookings (SD, USA). We used their primary diapausing strain, which was maintained at ~8 °C until application. The eggs were applied when the plants reached the V2 stage as described above. Eggs were mixed into a solution of water containing agar at the final concentration of 0.15%, and each plant was exposed to ~800 viable eggs delivered evenly down both sides of the row with a tractor-mounted system. The number of replicates for each treatment in the experiment carried out in 2015 was 8, for a total of 96 experimental plots, while in field experiments performed in 2016 and 2017 the experiment was doubled to facilitate data collection, for a total of 192 experimental plots (Supplementary Material 1). All replicates were arranged in a randomized complete block design with 8 blocks (each containing 12 treatments in a split-plot design WCR vs no WCR) for the field trial 2015, and with 16 blocks for the experiments carried out the following two years. The 16 blocks in 2016 and 2017 still resulted in 8 replications because half were used for damage plus larval recovery and half for yield.

### Evaluation of WCR damage severity and maize yield

About six weeks after the WCR eggs were applied, approximately 500 degree-days post infestation as calculated with the techniques of Hibbard *et al*.^67^, root damage was evaluated on three plants per plot. Maize plants were dug out from the soil, their roots were washed, and damage caused by WCR larval feeding was rated using the node injury score^5^.

We also evaluated the presence of WCR larvae on the roots. For this, two additional plants were removed from each plot at approximately 410 degree days^67^ post infestation, when most larvae should be at the early third instar. Following Hibbard *et al*.^68^, the entire root system of each collected plant was placed into onion bags and the bags were suspended in a greenhouse (38–50 °C). A water pan was positioned under each bag to collect all larvae that fell down. Larvae were collected and counted until no additional larvae were recovered for three consecutive days. To estimate the impact of the different treatments on the WCR fitness, collected larvae were counted and weighed.

At the end of the season, maize cobs from the three remaining plants per plot (2015) or from the yield portion of the study were harvested and grain yield was determined and expressed in total grain weight.

### Monitoring of beneficial organisms

#### *Pseudomonas* bacteria

In 2017, we monitored the presence of the *Pseudomonas* strains in the different plots. Maize roots were sampled about 5 weeks after the application of WCR eggs. For this, the root systems from six maize plants (i.e. two plants taken from three extra-plots specifically planted to assess *Pseudomonas* survival during the field experiment) were dug up, pooled, washed and gently dried using paper towels. To avoid cross-contamination between samples, all material used for the sampling at the field site was cleaned with 70% ethanol. Roots were placed in 15 ml sterile conical screw cap centrifuge tubes (Basix) containing 40 ml of sterile water and vigorously agitated on a rotary shaker at 180 rpm for 15–20 min. Subsequently roots were removed from the tubes, dried at 80 °C for three days and weighed. The remaining suspensions were transferred to fresh sterile tubes on ice and centrifuged at 8500 rpm (9300 g) at 4 °C. The obtained pellet was re-suspended in 1 ml of sterile water. Each sample was then serially diluted and dilutions plated on LB Agar Miller containing 100 μg/ml of cycloheximide (Sigma-Aldrich) and 100 μg/ml of rifampicin^69^. The colonies were counted and the results were expressed as colony forming units (CFU) per gram of dry root weight.

#### Entomopathogenic nematodes

In 2017, soil samples were taken from each of the plots inoculated with the EPN mix. Approximately 2,000 cm^3^ of soil was sampled from the plots near the plants by taking multiple scoops approximately 12 cm deep into the soil. Individual plot samples were mixed and two subsamples of approximately 120 ml were placed into 236 ml plastic containers (Solo Cup Company, Lake Forest, IL, USA) and baited with two last-instar *Galleria mellonella* L. (Lepidoptera: Pyralidae) larvae each. Samples were maintained in the dark at 20 °C and checked daily for *G. mellonella* mortality. If cadavers were found with nematodes present as typical EPN symptom^70^, the plots tested were recorded as having an active nematode population.

### Statistical analysis

All statistical analyses were performed using the software package R^71^, version 3.2.3. Data were checked for normal distribution with the Shapiro-Wilk test and by plotting QQ-Plots. Equality of variance was verified performing Bartlett’s test. Most of the data failed the normality and equality of variance assumptions, therefore non-parametric Kruskal-Wallis analysis of variance on ranks (H-tests) were carried out. Post-hoc test analyses were conducted using Fisher’s least significant difference with a Benjamini-Hochberg correction of *P*-values (package “agricolae”)^72^. Results obtained in the control experiment in which the carrier substrate for the AMF was tested alone (AMF “mock” inoculum) were not significantly different from those obtained in the untreated control. Therefore, the control and AMF-control were pooled to facilitate the interpretation of the results. Moreover, the effect of the WCR infestation (infested *versus* non-infested) was so high compared to the effect of the beneficial organisms treatments, that, in order to detect differences among application treatments, the effect of the WCR infestation was assessed separately.

## Results

### Impact of beneficial soil organisms’ application on maize root damage

**Figure 1:**
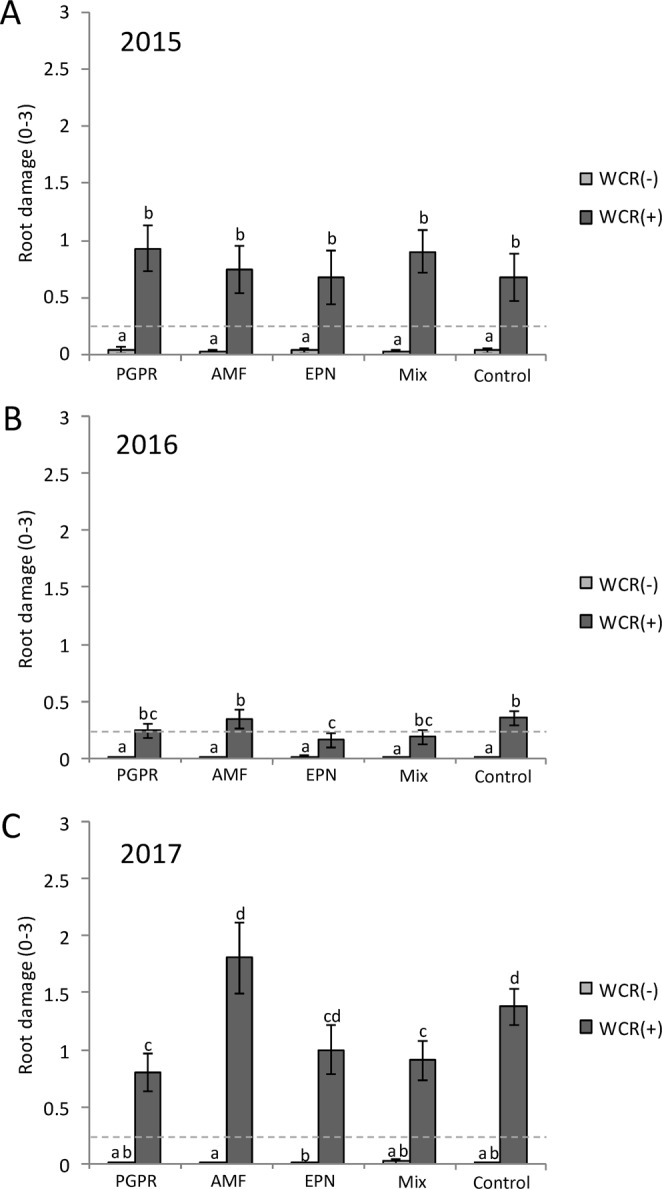
Root damage measured on the node-injury scale (Oleson *et al*.^5^) depending on the beneficial organisms applied and the western corn root worm (WCR) infestation status. (**A**) in 2015, (**B**) in 2016 and (**C**) in 2017. The dash line represents the economical threshold of root damage. PGPR: plant-growth promoting rhizobacteria, EPN: entomopathogenic nematodes, AMF: arbuscular mycorrhizial fungi, Mix: PGPR + EPN + AMF. Bars represent mean percentage ± SE. Means denoted by different letters are significantly different (*P* < 0.05, Fisher’s least significant difference test).

In 2015, all plots that were artificially infested with WCR showed significantly more damage than non-infested plots, revealing the efficiency of the infestation (Chisq = 53.65, *P* < 0.001). Average damage was 0.78 on the Oleson scale for infested plots (Fig. 1A). The application of the beneficial soil organisms did not reduce the damage caused by WCR larvae to maize roots (Fig. 1A). For the 2016 field trial, average root damage in the infested plots was 0.25 on the Oleson scale, which was again significantly greater than in non-infested plots (Chisq = 63.8, *P* < 0.001) (Fig. 1B). Maize roots from plots treated with PGPR, EPN and the combination of PGPR, EPN and AMF (Mix), were slightly less damaged compared to untreated plants, although the observed difference was statistically significant only for the EPN treatment, which showed significantly less root damage than the control plots (*P* = 0.03). Maize roots were most damaged in plots treated with AMF, and data were not significantly different compared to the control plots (*P* = 0.88) (Fig. 1B). In 2017, the average value of root damage was 1.18, the highest observed over the three consecutive years. Once again, all plots infested with WCR showed significantly more damage than non-infested plots (Chisq = 73.1, *P* < 0.001). Maize roots from plots treated with PGPR, EPN and the Mix were slightly less damaged as compared to untreated plants. The observed differences were statistically significant for the PGPR and the Mix treatment as compared to the AMF and the control plots (PGPR: AMF, *P* = 0.01; PGPR: control, *P* = 0.02; Mix: AMF, *P* = 0.02; Mix: control, *P* = 0.05) (Fig. 1C).

### Impact of treatments on WCR survival and weight

**Figure 2:**
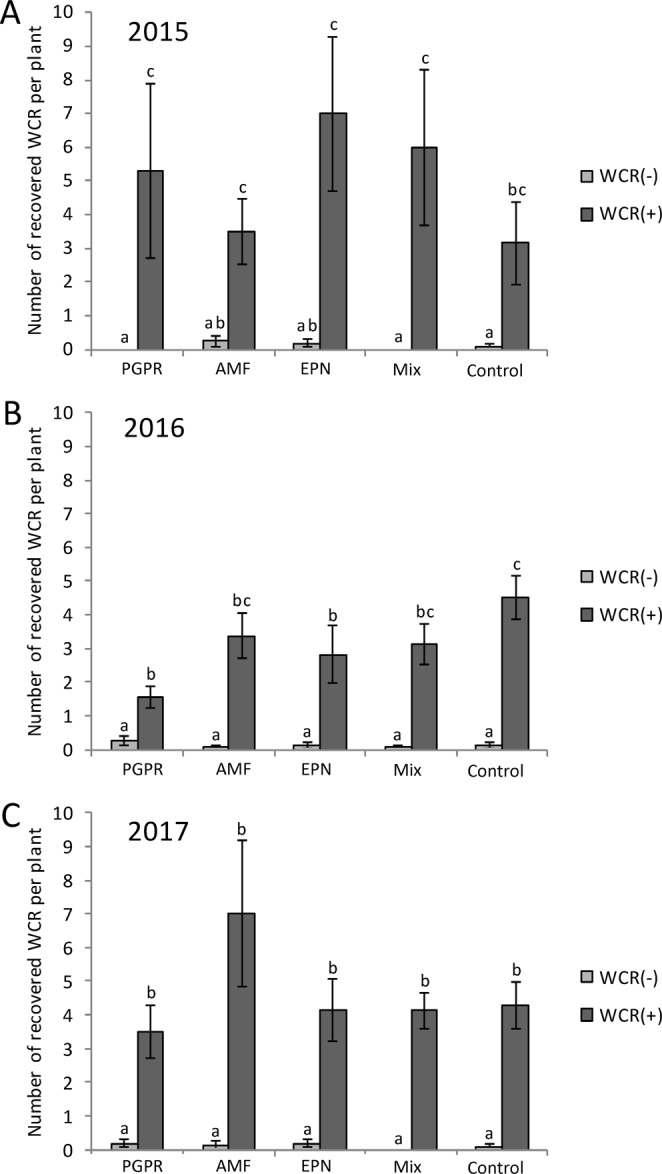
Number of western corn root worm larvae recovered from root system depending on the beneficial organisms applied and the western corn root worm (WCR) infestation status. (**A**) In 2015, (**B**) in 2016 and (**C**) in 2017. PGPR: plant-growth promoting rhizobacteria, EPN: entomopathogenic nematodes, AMF: arbuscular mycorrhizial fungi, Mix: PGPR + EPN + AMF. Bars represent mean percentage ± SE. Means denoted by different letters are significantly different (*P* < 0.05, Fisher’s least significant difference test).

Almost no WCR larvae were recovered from non-infested plots as compared to infested plots (2015: Chisq = 34.3, *P* < 0.001; 2016: Chisq = 68.4, *P* < 0.001; 2017: Chisq = 76.1, *P* < 0.001) (Fig. 2). In 2015, the number of recovered WCR larvae was not affected by the various soil applications, but tended to be slightly higher compared to the control plots (Fig. 2). In 2016, however, the number of recovered WCR larvae was significantly lower in plots with PGPR application and in plots with EPN application, but not in plots with the mixture containing the two plus AMF (PGPR: control, *P* = 0.003; EPN: control, *P* = 0.03 (Fig. 2B). In 2017, the beneficial soil organisms did not negatively affect the number of recovered WCR larvae (Fig. 2C).

**Figure 3:**
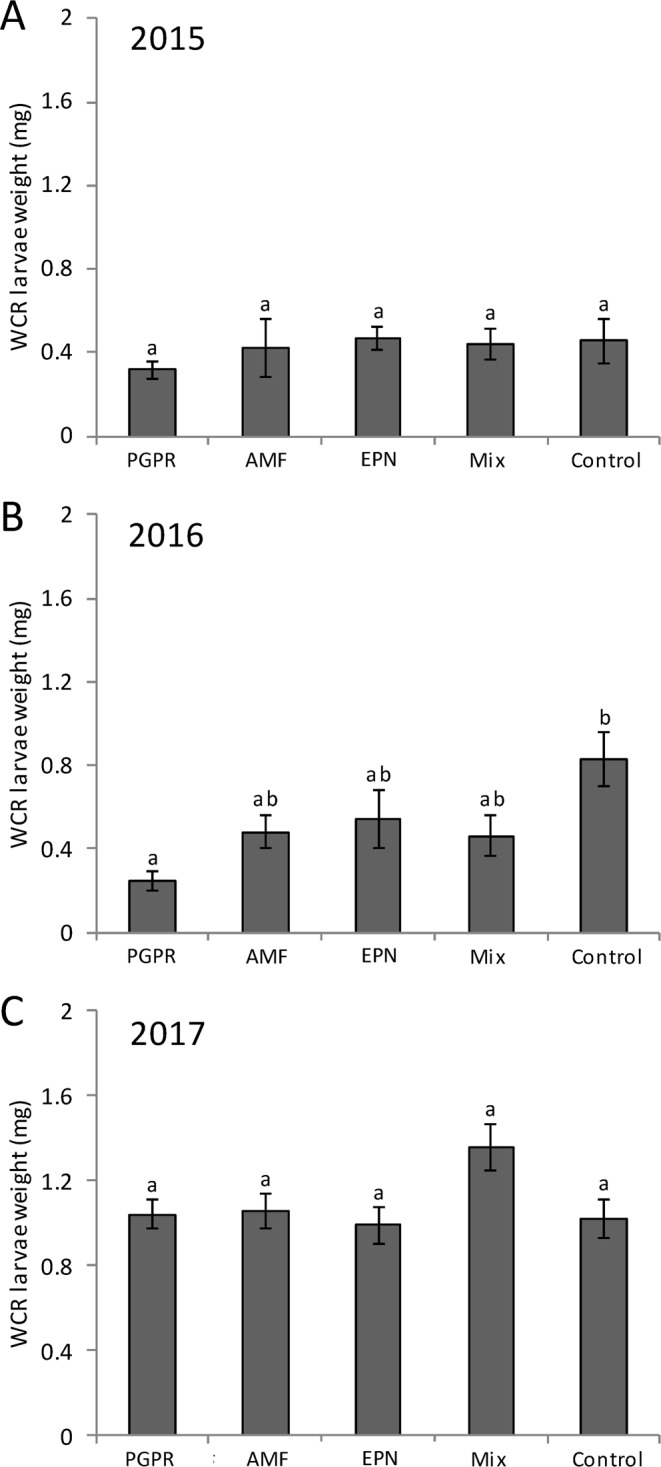
Western corn rootworm weight in response to the application of beneficial organisms. (**A**) In 2015, (**B**) in 2016 and (**C**) in 2017. PGPR: plant-growth promoting rhizobacteria, EPN: entomopathogenic nematodes, AMF: arbuscular mycorrhizial fungi, Mix: PGPR + EPN + AMF. Bars represent mean percentage ± SE. Means denoted by different letters are significantly different (*P* < 0.05, Fisher’s least significant difference test).

In 2015, WCR larvae weight was not affected by the applications (Fig. 3A). In 2016, WCR larvae in PGPR plots weight significantly less compared to those recovered from control plots (PGPR: control, *P* = 0.02), while no significant differences were observed for the other treatments (Fig. 3B). In 2017, no differences in larval weight were observed among the different treatment plots (Fig. 3C).

### Impact of treatments on yield

**Figure 4:**
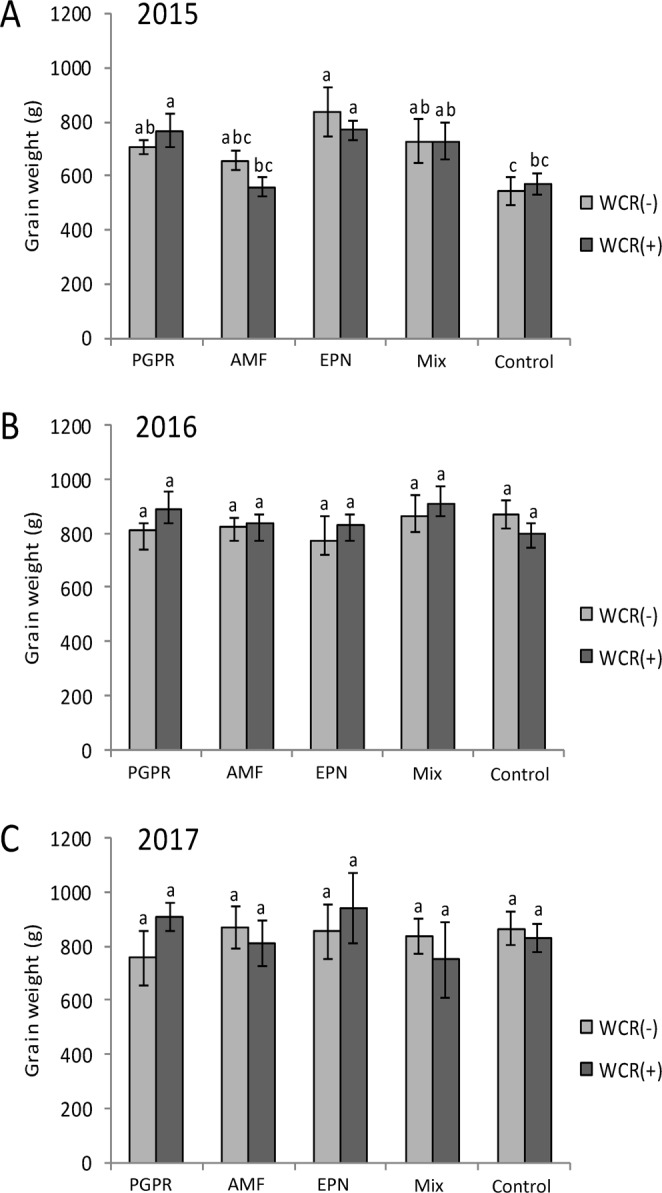
Yield, expressed as maize grain weight, in response to the application of beneficial organisms and the western corn root worm (WCR) infestation status. (**A**) in 2015, (**B**) in 2016 and (**C**) in 2017. PGPR: plant-growth promoting rhizobacteria, EPN: entomopathogenic nematodes, AMF: arbuscular mycorrhizial fungi, Mix: PGPR + EPN + AMF. Bars represent mean percentage ± SE. Means denoted by different letters are significantly different (*P* < 0.05, Fisher’s least significant difference test).

The infestation with WCR did not have any impact on yield (expressed as gram of seed per plot) in any of the field trials (2015: Chisq = 0.01, *P* = 0.9; 2016: Chisq = 0.1, *P* = 0.7; 2017: Chisq = 0.1, *P* = 0.7) (Fig. 4). However, in 2015, yield was positively impacted by PGPR and EPN applications (independently of the WCR infestation) (PGPR: control, *P* = 0.01, EPN: control, *P* = 0.01). In 2016 and 2017, the beneficial soil organisms had no impact on yield (Fig. 4B,C).

### Persistence of the applied rhizobacteria and nematodes

In 2017, nematodes were found in all of the corresponding augmented plots. In plots that had been treated with rhizobacteria, the numbers of rifampicin-resistant *Pseudomonas* varied between 1.05 × 10^3^ and 3.81 × 10^5^ CFU.g^−1^ of dry root weight. No rifampicin-resistant bacteria were found in control plots.

## Discussion

Overall, our results confirm that PGPR and EPN can protect maize roots from WCR, as observed through a reduction in root damage in plots where they were applied separately or in combination with AMF. In 2016, in plots treated with EPNs, root damage was reduced below the economic threshold. This was expected because both EPN species used in this study (*S. feltiae* and *H. bacteriophora*) are known to readily kill WCR^23–26,73–75^, and in a previous study on the same experimental farm, root damage by WCR was reduced by the application of a slightly lower dose of *H. bacteriophora* (50 IJs/cm^2^)^76^. From our 2017 trial, we can conclude that PGPR application can also significantly reduced WCR-inflicted root damage. For the PGPR, the observed reduction of root feeding may be explained by induced systemic resistance^77,78^, as well as by direct insecticidal effects. Ours is the first field trial to test if *Pseudomonas* strains application can reduce WCR pressures. Yet, from a study using transgenic maize plants expressing an insecticidal protein that is naturally produced by a *P. chlororaphis* isolate, it is known that it strongly affects WCR feeding and survival^79^.

PGPR application in the 2016 trial was the only treatment that reduced WCR weight, possibly explained by enhanced plant defense or increased infection of the insects. In 2016 and 2017, the yield was not affected by WCR infestation, nor by any of the applications of soil organisms. Apparently, despite significant damage to the root system, the plants were able to somehow compensate and still be fully productive^80^. Although PGPR and EPN did not have any detectable impact on WCR in 2015, this was the only year where yield was increased following their application. Impact of WCR was minimal during 2015, probably due to waterlogging of the plots. We speculate that the positive impact of EPN and PGPR application may have been the result of induced resistance against pathogens and growth promotion, which are known properties of these organisms^77,78,81,82^. For instance, *Pseudomonas* spp. produce antimicrobial compounds that can stimulate systemic resistance in plants^82^, or act as growth promoters or inhibitors and increase stress tolerance^83^. EPN have also been shown to induce such resistance in plants, but the mechanisms that are involved remain to be elucidated^81^. Also, phytohormones like auxin, cytokinin, gibberellin or ethylene of microbial and fungal origin can affect growth, root development, immune response and hormonal pathways in plants^38,51^. PGPR are also involved in the solubilization of mineral phosphates and other nutrients that can facilitate their access by the plant^84,85^. We should stress that the bacterial strains used in 2015 (i.e., *P. protegens* Pf-5 and *P. chlororaphis* O6) were not the same as those used in 2016 and 2017 (i.e., *P. protegens* CHA0 and *P. chlororaphis* PCL1391) and, although they were very similar, possible differences between strains may have had an impact on their effectiveness. All four strains possess the cluster *fit* that directs the synthesis of FitD, the insecticidal protein that enables these bacteria to kill different insect pests^53,54^.

The application of AMF, which were included to confirm their compatibility with the nematodes and bacteria, did not significantly affect WCR survival and performance, nor did it affect plant performance. AMF were applied before the development of the first roots because the spores can persist for a long time in the soil until they form hyphae to colonize roots^86^. Unfortunately, for practical reasons, we could not assess the establishment success of the AMF, but the same commercial inoculum is commonly used to enhance crop performance and has been shown to successfully colonize rice roots^87^. It is, however, possible that our inoculant was not effective and failed to persist in the soil and to colonize roots, as was reported for another trail with similar AMF species for arable maize^88^. From a Swiss study in wheat plots with a different AMF inoculum we know that after application at the seedling stage, it has the potential to persist and can successfully colonize the roots^59^. Although we can safely conclude that the applied AMF had no effect on the efficacy of the PGPR and EPN, for future studies, it would be desirable to gather information on the inoculant fate over the field seasons to confirm persistence and colonization status.

We observed no synergistic or additive effect of the soil organisms as their combined application did not result in a higher efficacy to protect maize roots from damage or increase yield. Therefore, it is not excluded that interactions between the applied soil organisms in some ways limit their full potential. The weather, and therefore the field conditions, were very different from one year to another (Supplementary Material 2), in particular in terms of precipitation, which surely affected the results. To compare the weather over the entire period of each trial, we took into account several parameters from 1^st^ May to 1^st^ November, for each year. Rainfall, with 625 mm, was intermediate for 2015, whereas 2016 was wetter (700 mm) and 2017 much drier (506 mm). Temperature was quite stable with an average of 20.5 °C, 21.1 °C, and 19.9 °C for 2015, 2016 and 2017, respectively. Based on these values we can assume that soil moisture was quite different among years and this must have had a significant impact on the organisms that we applied to the field. For EPNs, soil moisture determines the thickness of the water film that the nematodes need to move and survive, and it also affects the surface tension and the amount of oxygen present in the soil. These parameters influence the efficacy and survival of EPN^89^ and could explain why EPN application was most effective in 2016, the year with the highest precipitation rate. Soil moisture is also one of the best predictors of soil microbial biomass: wet soils normally contain a greater bacterial biomass than dry soils^90^. Yet, Burr *et al*.^91^ found that specific strains of *P. fluorescens* and *P. putida* were able to persist under field conditions for many weeks when the soil was “relatively dry”. Soil moisture was also a key factor in a field study on stress tolerance of *P. protegens* Pf-5^59^. Interestingly, the level of irrigation can have contrasting effects on the abundance of different *Pseudomonas* strains. For instance, strains that produce the antimicrobial compounds 2,4-diacetylphloroglucinol are more present in irrigated soils, whereas phenazines producers are more abundant in drier terrains^92^. Soil moisture effects on the persistence and performance of the strains used in our study have not yet been specifically tested.

In this study we chose to apply the soil organisms at seeding as a strategy to reduce the field work-load, using a single event for seeding and the application of the biocontrol agents. The infestation with WCR eggs occurred about two weeks after the application of the soil organisms and the WCR larvae started feeding on the roots about three weeks after application. Therefore, the soil organisms must persist in the soil for this period of time to have an effect. Applying the soil organisms after WCR infestation can be expected to be more effective in controlling the pest, but would be much more labor intensive.

## Conclusion

As is often the case with field studies, the results were quite different for the different years. Yet, each year at least one of the treatments was significantly better compared to the control (Figs 1–3). Depending on the year, the treatments had a direct impact on corn plant performance, but also impacted the survival and performance of the WCR larvae. The results obtained in 2016 were particularly encouraging, although the effect on WCR was not as evident as the two other two years. The fact that the yield was not been significantly compromised by the artificial WCR infestation explains at least partially why we found no significant impact on plant productivity, except for 2015. During years 2016 and 2017, the level of root damage proved to be a suitable parameter to measure treatment effects. We think that studies such as this one can be the basis for the development of effective soil treatments that can replace the use of pesticides, and provide a more sustainable control of WCR and other soil pests.

## Supplementary information

Supplementary information

Supplementary dataset

## Data Availability

The datasets generated during and/or analyzed during the current study are provided in the supplementary materials.
