# Supplementary material for: Protecting maize from rootworm damage with the combined application of arbuscular mycorrhizal fungi, Pseudomonas bacteria and entomopathogenic nematodes
Source: Sci Rep. 2019 Feb 28;9:3127. doi: 10.1038/s41598-019-39753-7 (PMC6395644; doi:10.1038/s41598-019-39753-7)
Supplement: Supplementary file 1 — Supplementary information [file 41598_2019_39753_MOESM1_ESM.pdf]

Protecting maize from rootworm damage with the combined application of arbuscular mycorrhizal fungi, *Pseudomonas* bacteria and entomopathogenic nematodes

Nicola Imperiali[+1], Geoffrey Jaffuel[+2], Kent Shelby[3], Raquel Campos-Herrera[2,4], Ryan Geisert[3], Monika Maurhofer[5], Joyce Loper[6,7], Christoph Keel[1], Ted. C.J Turlings[2], Bruce E. Hibbard[8]

[1]Department of Fundamental Microbiology, University of Lausanne, Lausanne, Switzerland

[2]FARCE Laboratory, Institute of Biology, University of Neuchâtel, Neuchâtel, Switzerland

[3]Biological Control of Insects Research, US Department of Agriculture, Agricultural Research Service, Columbia, MO, USA

[4]Instituto de Ciencias de la Vid y del Vino, CSIC-Universidad de La Rioja-Gobierno de La Rioja, Logroño, Spain

[5]Institute of Integrative Biology, ETH Zurich, Zurich, Switzerland

[6]Department of Botany and Plant Pathology, Oregon State University, Corvallis, OR, USA

[7]Horticultural Crops Research Laboratory, US Department of Agriculture, Agricultural Research Service, Corvallis, OR, USA

[8]Plant Genetics Research Unit, US Department of Agriculture-ARS, University of Missouri, Columbia, MO, USA

[+]These authors contributed equally to the work

Correspondence: T. Turlings & B. Hibbard; email: ted.turlings@unine.ch & Bruce.Hibbard@ars.usda.gov

Supplementary material 1:

Field plot design

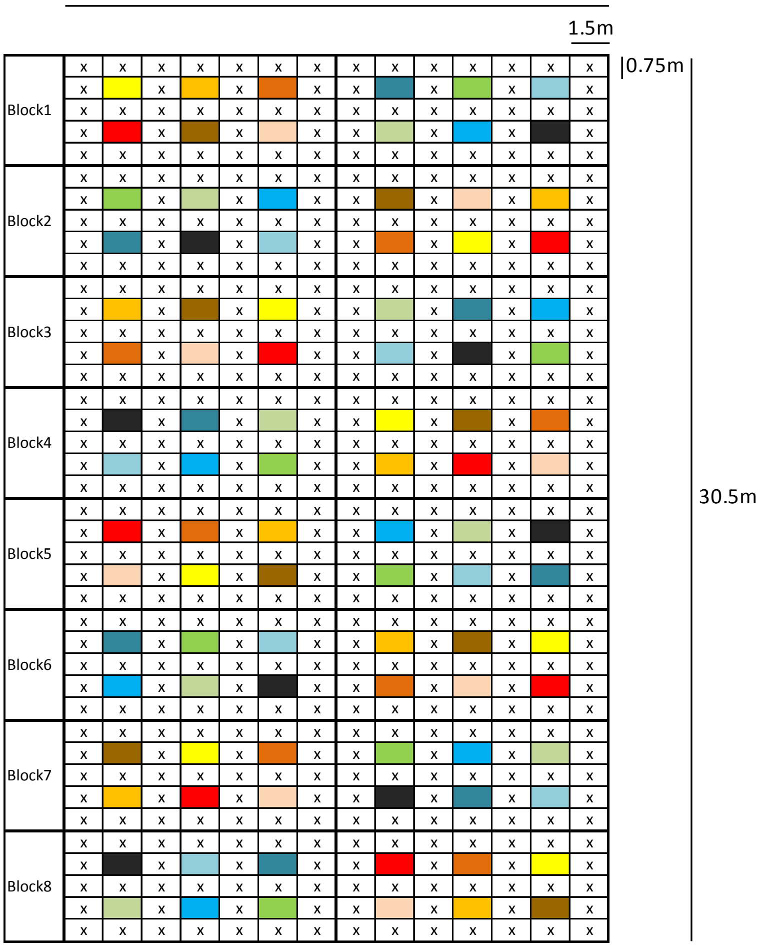

WCR infestation	Benefical organism	Color code
(-)	Bacteria	
(-)	Fungi	
(-)	Nematode	
(-)	Mix	
(-)	Control	
(-)	Control-Fungi	
(+)	Bacteria	
(+)	Fungi	
(+)	Nematode	
(+)	Mix	
(+)	Control	
(+)	Control-Fungi	

Experimental design for the field trial conducted in 2015, resulting in 8 blocks of 12 plots each, for a total of 96 plots. In 2016 and 2017, the size of the experiments was doubled, with 16 blocks of 12 plots each, for a total of 192 plots.

Supplementary Material 2

Field trials (year)	2015	2016	2017
**Parameters recorded from 1st May to 1st November**			
Total precipitation (mm)	625.11	700.24	506.22
Average maximum air temperature (˚C)	26.2	27.0	26.1
Average minimum air temperature (˚C)	15.3	15.7	14.0
Average air temperature (˚C)	20.5	21.1	19.9
Total solar radiation (MJ / m^2)	17.41	17.97	18.81
Vapor pressure (kPa)	1.884	1.958	1.749
Average maximum wind speed (m/s)	8.6	8.2	8.8

Main physical parameters recorded between the months of May and November in 2015, 2016 and 2017, **at the Bradford Research and Extension Centre**, **Columbia**, **Missouri**. Data were taken from the "Missouri Historical Agricultural Weather Database" platform. (http://agebb.missouri.edu/weather/history).
