# Supplementary material for: Protecting maize from rootworm damage with the combined application of arbuscular mycorrhizal fungi, Pseudomonas bacteria and entomopathogenic nematodes
Source: Sci Rep. 2019 Feb 28;9:3127. doi: 10.1038/s41598-019-39753-7 (PMC6395644; doi:10.1038/s41598-019-39753-7)
Supplement: Supplementary file 2 — Supplementary dataset [file 41598_2019_39753_MOESM2_ESM.pdf]

Protecting maize from rootworm damage with the combined application of arbuscular mycorrhizal fungi, *Pseudomonas* bacteria and entomopathogenic nematodes

Geoffrey Jaffuel[+1], Nicola Imperiali[+2], Kent Shelby[3], Raquel Campos-Herrera[1,4], Ryan Geisert[3], Monika Maurhofer[5], Joyce Loper[6,7], Christoph Keel[1], Ted. C.J Turlings[2], Bruce E. Hibbard[8]

[1]FARCE Laboratory, Institute of Biology, University of Neuchâtel, Neuchâtel, Switzerland

[2]Department of Fundamental Microbiology, University of Lausanne, Lausanne, Switzerland

[3]Biological Control of Insects Research, US Department of Agriculture, Agricultural Research Service, Columbia, MO, USA

[4]Instituto de Ciencias de la Vid y del Vino, CSIC-Universidad de La Rioja-Gobierno de La Rioja, Logroño, Spain

[5]Institute of Integrative Biology, ETH Zurich, Zurich, Switzerland

[6]Department of Botany and Plant Pathology, Oregon State University, Corvallis, OR, USA

[7]Horticultural Crops Research Laboratory, US Department of Agriculture, Agricultural Research Service, Corvallis, OR, USA

[8]Plant Genetics Research Unit, US Department of Agriculture-ARS, University of Missouri, Columbia, MO, USA

[+]These authors contributed equally to the work

Correspondence: T. Turlings & B. Hibbard; email: ted.turlings@unine.ch &Bruce.Hibbard@ars.usda.gov

Field design 2015

Block	Treat-Number	WCR	Beneficial	Missouri Plot Number
1	**7**	YES	Bacteria	1
1	**9**	YES	Nematode	2
1	**8**	YES	Fungi	3
1	**3**	NO	Nematode	4
1	**2**	NO	Fungi	5
1	**1**	NO	Bacteria	6
1	**10**	YES	Mix	7
1	**11**	YES	Control	8
1	**12**	YES	Control-Fungi	9
1	**6**	NO	Control-Fungi	10
1	**5**	NO	Control	11
1	**4**	NO	Mix	12
2	**2**	NO	Fungi	13
2	**6**	NO	Control-Fungi	14
2	**5**	NO	Control	15
2	**11**	YES	Control	16
2	**12**	YES	Control-Fungi	17
2	**9**	YES	Nematode	18
2	**3**	NO	Nematode	19
2	**4**	NO	Mix	20
2	**1**	NO	Bacteria	21
2	**8**	YES	Fungi	22
2	**7**	YES	Bacteria	23
2	**10**	YES	Mix	24
3	**9**	YES	Nematode	25
3	**11**	YES	Control	26
3	**7**	YES	Bacteria	27
3	**6**	NO	Control-Fungi	28
3	**3**	NO	Nematode	29
3	**5**	NO	Control	30
3	**8**	YES	Fungi	31
3	**12**	YES	Control-Fungi	32
3	**10**	YES	Mix	33
3	**1**	NO	Bacteria	34
3	**4**	NO	Mix	35
3	**2**	NO	Fungi	36
4	**4**	NO	Mix	37
4	**3**	NO	Nematode	38
4	**6**	NO	Control-Fungi	39
4	**7**	YES	Bacteria	40
4	**11**	YES	Control	41
4	**8**	YES	Fungi	42
4	**1**	NO	Bacteria	43
4	**5**	NO	Control	44
4	**2**	NO	Fungi	45
4	**9**	YES	Nematode	46
4	**10**	YES	Mix	47

4	**12**	YES	Control-Fungi	48
5	**10**	YES	Mix	49
5	**8**	YES	Fungi	50
5	**9**	YES	Nematode	51
5	**5**	NO	Control	52
5	**6**	NO	Control-Fungi	53
5	**4**	NO	Mix	54
5	**12**	YES	Control-Fungi	55
5	**7**	YES	Bacteria	56
5	**11**	YES	Control	57
5	**2**	NO	Fungi	58
5	**1**	NO	Bacteria	59
5	**3**	NO	Nematode	60
6	**3**	NO	Nematode	61
6	**2**	NO	Fungi	62
6	**1**	NO	Bacteria	63
6	**9**	YES	Nematode	64
6	**11**	YES	Control	65
6	**7**	YES	Bacteria	66
6	**5**	NO	Control	67
6	**6**	NO	Control-Fungi	68
6	**4**	NO	Mix	69
6	**8**	YES	Fungi	70
6	**12**	YES	Control-Fungi	71
6	**10**	YES	Mix	72
7	**11**	YES	Control	73
7	**7**	YES	Bacteria	74
7	**8**	YES	Fungi	75
7	**2**	NO	Fungi	76
7	**5**	NO	Control	77
7	**6**	NO	Control-Fungi	78
7	**9**	YES	Nematode	79
7	**10**	YES	Mix	80
7	**12**	YES	Control-Fungi	81
7	**4**	NO	Mix	82
7	**3**	NO	Nematode	83
7	**1**	NO	Bacteria	84
8	**4**	NO	Mix	85
8	**1**	NO	Bacteria	86
8	**3**	NO	Nematode	87
8	**10**	YES	Mix	88
8	**8**	YES	Fungi	89
8	**7**	YES	Bacteria	90
8	**6**	NO	Control-Fungi	91
8	**5**	NO	Control	92
8	**2**	NO	Fungi	93
8	**12**	YES	Control-Fungi	94
8	**9**	YES	Nematode	95
8	**11**	YES	Control	96

Root damage 2015

Plot	Beneficial	WCR	Damage (1-3)
2006	Bacteria	NO	0.005
2021	Bacteria	NO	0.025
2034	Bacteria	NO	0.0125
2043	Bacteria	NO	0.0625
2059	Bacteria	NO	0
2063	Bacteria	NO	0
2084	Bacteria	NO	0
2086	Bacteria	NO	0.1875
2005	Fungi	NO	0
2013	Fungi	NO	0.025
2036	Fungi	NO	0.0625
2045	Fungi	NO	0
2058	Fungi	NO	0
2062	Fungi	NO	0
2076	Fungi	NO	0.0675
2093	Fungi	NO	0.005
2004	Nematode	NO	0
2019	Nematode	NO	0
2029	Nematode	NO	0
2038	Nematode	NO	0.0675
2060	Nematode	NO	0
2061	Nematode	NO	0.125
2083	Nematode	NO	0.025
2087	Nematode	NO	0.0375
2012	Mix	NO	0
2020	Mix	NO	0.025
2035	Mix	NO	0
2037	Mix	NO	0.025
2054	Mix	NO	0.0625
2069	Mix	NO	0
2082	Mix	NO	0.0375
2085	Mix	NO	0.05
2011	Control	NO	0.125
2077	Control	NO	0
2015	Control	NO	0
2030	Control	NO	0
2052	Control	NO	0.005
2092	Control	NO	0.066666667
2044	Control	NO	0
2067	Control	NO	0
2010	Control-Fungi	NO	0.275
2014	Control-Fungi	NO	0
2028	Control-Fungi	NO	0.033333333
2039	Control-Fungi	NO	0.006666667
2053	Control-Fungi	NO	0
2068	Control-Fungi	NO	0.005
2078	Control-Fungi	NO	0

2091	Control-Fungi	NO	0.0025
2001	Bacteria	YES	0.6875
2023	Bacteria	YES	0.0025
2027	Bacteria	YES	0.5625
2040	Bacteria	YES	0.5
2056	Bacteria	YES	1.3125
2066	Bacteria	YES	1.5625
2074	Bacteria	YES	1.1875
2090	Bacteria	YES	1.625
2003	Fungi	YES	0.833333333
2022	Fungi	YES	0
2031	Fungi	YES	1.6875
2042	Fungi	YES	1.125
2050	Fungi	YES	1.125
2070	Fungi	YES	0.1375
2075	Fungi	YES	0.1925
2089	Fungi	YES	0.8125
2002	Nematode	YES	0.025
2018	Nematode	YES	1.5
2025	Nematode	YES	1.416666667
2046	Nematode	YES	0.04
2051	Nematode	YES	0.075
2064	Nematode	YES	0.0875
2079	Nematode	YES	1.25
2095	Nematode	YES	1
2007	Mix	YES	0.9375
2024	Mix	YES	1.625
2033	Mix	YES	0.275
2047	Mix	YES	0.003333333
2049	Mix	YES	0.875
2072	Mix	YES	1.125
2080	Mix	YES	0.9375
2088	Mix	YES	1.4375
2057	Control	YES	0.1375
2008	Control	YES	0.175
2065	Control	YES	1.333333333
2041	Control	YES	0.0775
2026	Control	YES	0.255
2016	Control	YES	0.09
2073	Control	YES	0.625
2096	Control	YES	1.125
2009	Control-Fungi	YES	0.5875
2017	Control-Fungi	YES	0.0125
2032	Control-Fungi	YES	1.3125
2048	Control-Fungi	YES	1.5625
2055	Control-Fungi	YES	1.75
2071	Control-Fungi	YES	0.275
2081	Control-Fungi	YES	0.325
2094	Control-Fungi	YES	1.166666667

Larval recovery 2015

Plot	Beneficial	WCR	larval recovery (mean nb)
2001	Bacteria	YES	0.5
2023	Bacteria	YES	0
2027	Bacteria	YES	0
2040	Bacteria	YES	0
2056	Bacteria	YES	11.5
2066	Bacteria	YES	5.5
2074	Bacteria	YES	4.5
2090	Bacteria	YES	20.5
2006	Bacteria	NO	0
2021	Bacteria	NO	0
2034	Bacteria	NO	0
2043	Bacteria	NO	0
2059	Bacteria	NO	0
2063	Bacteria	NO	0
2084	Bacteria	NO	0
2086	Bacteria	NO	0
2008	Control	YES	0
2016	Control	YES	2
2026	Control	YES	8
2041	Control	YES	0
2057	Control	YES	0
2065	Control	YES	0
2073	Control	YES	4
2096	Control	YES	10.5
2011	Control	NO	0
2015	Control	NO	0
2030	Control	NO	0
2044	Control	NO	0
2052	Control	NO	1
2067	Control	NO	0.5
2077	Control	NO	0
2092	Control	NO	0
2009	Control-Fungi	YES	7
2017	Control-Fungi	YES	0
2032	Control-Fungi	YES	1
2048	Control-Fungi	YES	1.5
2055	Control-Fungi	YES	16.5
2071	Control-Fungi	YES	0
2081	Control-Fungi	YES	0
2094	Control-Fungi	YES	0
2010	Control-Fungi	NO	0
2014	Control-Fungi	NO	0
2028	Control-Fungi	NO	0
2039	Control-Fungi	NO	0
2053	Control-Fungi	NO	0
2068	Control-Fungi	NO	0
2078	Control-Fungi	NO	0

2091	Control-Fungi	NO	0
2003	Fungi	YES	1.5
2022	Fungi	YES	0
2031	Fungi	YES	5.5
2042	Fungi	YES	4
2050	Fungi	YES	7.5
2070	Fungi	YES	4
2075	Fungi	YES	0
2089	Fungi	YES	5.5
2005	Fungi	NO	0
2013	Fungi	NO	1
2036	Fungi	NO	0
2045	Fungi	NO	0
2058	Fungi	NO	0
2062	Fungi	NO	1
2076	Fungi	NO	0
2093	Fungi	NO	0
2007	Mix	YES	4.5
2024	Mix	YES	2
2033	Mix	YES	1
2047	Mix	YES	0
2049	Mix	YES	8
2072	Mix	YES	18
2080	Mix	YES	13
2088	Mix	YES	1.5
2012	Mix	NO	0
2020	Mix	NO	0
2035	Mix	NO	0
2037	Mix	NO	0
2054	Mix	NO	0
2069	Mix	NO	0
2082	Mix	NO	0
2085	Mix	NO	0
2002	Nematode	YES	0
2018	Nematode	YES	18
2025	Nematode	YES	11
2046	Nematode	YES	0
2051	Nematode	YES	8.5
2064	Nematode	YES	0.5
2079	Nematode	YES	11
2095	Nematode	YES	7
2004	Nematode	NO	0.5
2019	Nematode	NO	0
2029	Nematode	NO	0
2038	Nematode	NO	0
2060	Nematode	NO	0
2061	Nematode	NO	1
2083	Nematode	NO	0
2087	Nematode	NO	0

Larval weigth 2015

Plot	Beneficial	WCR	larvae weight (mg)
2001	Bacteria	YES	1
2023	Bacteria	YES	0
2027	Bacteria	YES	0
2040	Bacteria	YES	0
2056	Bacteria	YES	11.5
2066	Bacteria	YES	5.5
2074	Bacteria	YES	4.5
2090	Bacteria	YES	20.5
2008	Control	YES	0
2016	Control	YES	4
2026	Control	YES	8
2041	Control	YES	0
2057	Control	YES	0
2065	Control	YES	0
2073	Control	YES	4
2096	Control	YES	10.5
2009	Control-Fungi	YES	14
2017	Control-Fungi	YES	0
2032	Control-Fungi	YES	1
2048	Control-Fungi	YES	1.5
2055	Control-Fungi	YES	16.5
2071	Control-Fungi	YES	0
2081	Control-Fungi	YES	0
2094	Control-Fungi	YES	0
2003	Fungi	YES	1.5
2022	Fungi	YES	0
2031	Fungi	YES	5.5
2042	Fungi	YES	4
2050	Fungi	YES	7.5
2070	Fungi	YES	4
2075	Fungi	YES	0
2089	Fungi	YES	5.5
2007	Mix	YES	4.5
2024	Mix	YES	2
2033	Mix	YES	1
2047	Mix	YES	0
2049	Mix	YES	8
2072	Mix	YES	18
2080	Mix	YES	26
2088	Mix	YES	1.5
2002	Nematode	YES	0
2018	Nematode	YES	18
2025	Nematode	YES	11
2046	Nematode	YES	0
2051	Nematode	YES	17
2064	Nematode	YES	1
2079	Nematode	YES	11

2095	Nematode	YES	7
2006	Bacteria	NO	0
2021	Bacteria	NO	0
2034	Bacteria	NO	0
2043	Bacteria	NO	0
2059	Bacteria	NO	0
2063	Bacteria	NO	0
2084	Bacteria	NO	0
2086	Bacteria	NO	0
2011	Control	NO	0
2015	Control	NO	0
2030	Control	NO	0
2044	Control	NO	0
2052	Control	NO	2
2067	Control	NO	0.5
2077	Control	NO	0
2092	Control	NO	0
2010	Control-Fungi	NO	0
2014	Control-Fungi	NO	0
2028	Control-Fungi	NO	0
2039	Control-Fungi	NO	0
2053	Control-Fungi	NO	0
2068	Control-Fungi	NO	0
2078	Control-Fungi	NO	0
2091	Control-Fungi	NO	0
2005	Fungi	NO	0
2013	Fungi	NO	2
2036	Fungi	NO	0
2045	Fungi	NO	0
2058	Fungi	NO	0
2062	Fungi	NO	2
2076	Fungi	NO	0
2093	Fungi	NO	0
2012	Mix	NO	0
2020	Mix	NO	0
2035	Mix	NO	0
2037	Mix	NO	0
2054	Mix	NO	0
2069	Mix	NO	0
2082	Mix	NO	0
2085	Mix	NO	0
2004	Nematode	NO	1
2019	Nematode	NO	0
2029	Nematode	NO	0
2038	Nematode	NO	0
2060	Nematode	NO	0
2061	Nematode	NO	2
2083	Nematode	NO	0
2087	Nematode	NO	0

Yield 2015

Plot	Beneficial	WCR	Weight (g)
2182	Bacteria	NO	600.7263288
2159	Bacteria	NO	640.1321616
2102	Bacteria	NO	664.2292536
2117	Bacteria	NO	676.136052
2139	Bacteria	NO	717.5263512
2130	Bacteria	NO	754.097232
2155	Bacteria	NO	761.4681072
2180	Bacteria	NO	849.068124
2140	Control	NO	319.4990904
2107	Control	NO	346.1476392
2111	Control	NO	346.9981248
2126	Control	NO	448.4894064
2173	Control	NO	517.6622352
2163	Control	NO	679.8214896
2188	Control	NO	686.6253744
2148	Control	NO	841.6972488
2135	Control-Fungi	NO	392.640852
2124	Control-Fungi	NO	403.6971648
2187	Control-Fungi	NO	415.6039632
2106	Control-Fungi	NO	438.000084
2164	Control-Fungi	NO	555.650592
2110	Control-Fungi	NO	572.3768088
2149	Control-Fungi	NO	701.9341152
2174	Control-Fungi	NO	1051.767192
2109	Fungi	NO	536.9399088
2172	Fungi	NO	564.4389432
2158	Fungi	NO	592.2214728
2189	Fungi	NO	601.5768144
2154	Fungi	NO	676.136052
2141	Fungi	NO	707.320524
2132	Fungi	NO	779.3283048
2101	Fungi	NO	806.2603488
2165	Mix	NO	362.5903608
2133	Mix	NO	552.5321448
2116	Mix	NO	622.271964
2178	Mix	NO	709.0214952
2131	Mix	NO	740.4894624
2181	Mix	NO	797.4719976
2108	Mix	NO	925.0448376
2150	Mix	NO	1113.28565
2183	Nematode	NO	551.1146688
2115	Nematode	NO	612.0661368
2134	Nematode	NO	679.8214896
2157	Nematode	NO	747.009852
2179	Nematode	NO	845.0991912
2125	Nematode	NO	898.1127936
2100	Nematode	NO	1047.798259

2156	Nematode	NO	1330.442974
2123	Bacteria	YES	372.2291976
2170	Bacteria	YES	735.1030536
2136	Bacteria	YES	744.1749
2152	Bacteria	YES	770.5399536
2119	Bacteria	YES	833.475888
2186	Bacteria	YES	868.3457976
2097	Bacteria	YES	908.3186208
2162	Bacteria	YES	912.854544
2104	Control	YES	422.9748384
2137	Control	YES	458.9787288
2122	Control	YES	511.708836
2169	Control	YES	599.8758432
2153	Control	YES	607.5302136
2112	Control	YES	633.611772
2161	Control	YES	657.9923592
2192	Control	YES	680.1049848
2128	Control-Fungi	YES	333.9573456
2190	Control-Fungi	YES	393.4913376
2177	Control-Fungi	YES	453.0253296
2167	Control-Fungi	YES	485.3437824
2105	Control-Fungi	YES	516.5282544
2151	Control-Fungi	YES	629.9263344
2113	Control-Fungi	YES	803.1419016
2144	Control-Fungi	YES	922.776876
2185	Fungi	YES	385.553472
2138	Fungi	YES	499.5185424
2118	Fungi	YES	531.5535
2146	Fungi	YES	553.6661256
2099	Fungi	YES	555.9340872
2127	Fungi	YES	579.1806936
2171	Fungi	YES	667.9146912
2166	Fungi	YES	699.6661536
2145	Mix	YES	477.4059168
2176	Mix	YES	555.650592
2129	Mix	YES	573.5107896
2103	Mix	YES	691.4447928
2184	Mix	YES	705.903048
2143	Mix	YES	843.39822
2120	Mix	YES	983.1613536
2168	Mix	YES	995.9186376
2121	Nematode	YES	625.3904112
2147	Nematode	YES	697.9651824
2098	Nematode	YES	700.233144
2160	Nematode	YES	738.504996
2191	Nematode	YES	769.9729632
2142	Nematode	YES	815.6156904
2175	Nematode	YES	851.903076
2114	Nematode	YES	961.3322232

Field design 2016

Plant damage/larval recovery plots

Block	Plot	Treatment	WCR		Beneficial	Code
1	3001	5	NO	1	Control	C
1	3002	4	NO	2	Mix	MIX
1	3003	3	NO	3	Nematode	N
1	3004	2	NO	4	Fungi	F
1	3005	1	NO	5	Bacteria	B
1	3006	6	NO	6	Control-Fungi	C-F
1	3007	9	YES	1	Nematode	N*
1	3008	10	YES	2	Mix	MIX*
1	3009	12	YES	3	Control-Fungi	C-F*
1	3010	11	YES	4	Control	C*
1	3011	7	YES	5	Bacteria	B*
1	3012	8	YES	6	Fungi	F*
2	3013	9	YES	1	Nematode	N*
2	3014	11	YES	2	Control	C*
2	3015	8	YES	3	Fungi	F*
2	3016	10	YES	4	Mix	MIX*
2	3017	12	YES	5	Control-Fungi	C-F*
2	3018	7	YES	6	Bacteria	B*
2	3019	2	NO	1	Fungi	F
2	3020	5	NO	2	Control	C
2	3021	6	NO	3	Control-Fungi	C-F
2	3022	1	NO	4	Bacteria	B
2	3023	3	NO	5	Nematode	N
2	3024	4	NO	6	Mix	MIX
3	3025	5	NO	1	Control	C
3	3026	6	NO	2	Control-Fungi	C-F
3	3027	3	NO	3	Nematode	N
3	3028	1	NO	4	Bacteria	B
3	3029	2	NO	5	Fungi	F
3	3030	4	NO	6	Mix	MIX
3	3031	9	YES	1	Nematode	N*
3	3032	12	YES	2	Control-Fungi	C-F*
3	3033	10	YES	3	Mix	MIX*
3	3034	11	YES	4	Control	C*
3	3035	7	YES	5	Bacteria	B*
3	3036	8	YES	6	Fungi	F*
4	3037	12	YES	1	Control-Fungi	C-F*
4	3038	11	YES	2	Control	C*
4	3039	9	YES	3	Nematode	N*
4	3040	8	YES	4	Fungi	F*
4	3041	10	YES	5	Mix	MIX*
4	3042	7	YES	6	Bacteria	B*
4	3043	6	NO	1	Control-Fungi	C-F
4	3044	5	NO	2	Control	C
4	3045	2	NO	3	Fungi	F
4	3046	4	NO	4	Mix	MIX

4	3047	**1**	NO	5	Bacteria	**B**
4	3048	**3**	NO	6	Nematode	**N**
5	3049	**11**	YES	1	Control	**C***
5	3050	**7**	YES	2	Bacteria	**B***
5	3051	**9**	YES	3	Nematode	**N***
5	3052	**8**	YES	4	Fungi	**F***
5	3053	**10**	YES	5	Mix	**MIX***
5	3054	**12**	YES	6	Control-Fungi	**C-F***
5	3055	**4**	NO	1	Mix	**MIX**
5	3056	**6**	NO	2	Control-Fungi	**C-F**
5	3057	**3**	NO	3	Nematode	**N**
5	3058	**5**	NO	4	Control	**C**
5	3059	**1**	NO	5	Bacteria	**B**
5	3060	**2**	NO	6	Fungi	**F**
6	3061	**4**	NO	1	Mix	**MIX**
6	3062	**3**	NO	2	Nematode	**N**
6	3063	**1**	NO	3	Bacteria	**B**
6	3064	**5**	NO	4	Control	**C**
6	3065	**2**	NO	5	Fungi	**F**
6	3066	**6**	NO	6	Control-Fungi	**C-F**
6	3067	**11**	YES	1	Control	**C***
6	3068	**9**	YES	2	Nematode	**N***
6	3069	**12**	YES	3	Control-Fungi	**C-F***
6	3070	**10**	YES	4	Mix	**MIX***
6	3071	**8**	YES	5	Fungi	**F***
6	3072	**7**	YES	6	Bacteria	**B***
7	3073	**5**	NO	1	Control	**C**
7	3074	**4**	NO	2	Mix	**MIX**
7	3075	**2**	NO	3	Fungi	**F**
7	3076	**1**	NO	4	Bacteria	**B**
7	3077	**6**	NO	5	Control-Fungi	**C-F**
7	3078	**3**	NO	6	Nematode	**N**
7	3079	**10**	YES	1	Mix	**MIX***
7	3080	**11**	YES	2	Control	**C***
7	3081	**7**	YES	3	Bacteria	**B***
7	3082	**9**	YES	4	Nematode	**N***
7	3083	**12**	YES	5	Control-Fungi	**C-F***
7	3084	**8**	YES	6	Fungi	**F***
8	3085	**11**	YES	1	Control	**C***
8	3086	**7**	YES	2	Bacteria	**B***
8	3087	**8**	YES	3	Fungi	**F***
8	3088	**9**	YES	4	Nematode	**N***
8	3089	**10**	YES	5	Mix	**MIX***
8	3090	**12**	YES	6	Control-Fungi	**C-F***
8	3091	**2**	NO	1	Fungi	**F**
8	3092	**1**	NO	2	Bacteria	**B**
8	3093	**6**	NO	3	Control-Fungi	**C-F**
8	3094	**5**	NO	4	Control	**C**
8	3095	**4**	NO	5	Mix	**MIX**
8	3096	**3**	NO	6	Nematode	**N**

Root damage 2016

Plot	Beneficial	WCR	Damage (1-3)
3005	Bacteria	NO	0.00666667
3022	Bacteria	NO	0.04
3028	Bacteria	NO	0
3047	Bacteria	NO	0
3059	Bacteria	NO	0
3063	Bacteria	NO	0.03333333
3076	Bacteria	NO	0
3092	Bacteria	NO	0
3004	Fungi	NO	0
3019	Fungi	NO	0
3029	Fungi	NO	0
3045	Fungi	NO	0
3060	Fungi	NO	0.04
3065	Fungi	NO	0
3075	Fungi	NO	0
3091	Fungi	NO	0.01666667
3003	Nematode	NO	0
3023	Nematode	NO	0
3027	Nematode	NO	0
3048	Nematode	NO	0.09
3057	Nematode	NO	0
3062	Nematode	NO	0
3078	Nematode	NO	0
3096	Nematode	NO	0
3002	Mix	NO	0
3024	Mix	NO	0
3030	Mix	NO	0
3046	Mix	NO	0
3055	Mix	NO	0
3061	Mix	NO	0
3074	Mix	NO	0.03333333
3095	Mix	NO	0
3001	Control	NO	0
3020	Control	NO	0
3025	Control	NO	0.00666667
3044	Control	NO	0
3058	Control	NO	0
3064	Control	NO	0.08333333
3073	Control	NO	0
3094	Control	NO	0.00666667
3006	Control-Fungi	NO	0.03333333
3021	Control-Fungi	NO	0
3026	Control-Fungi	NO	0
3043	Control-Fungi	NO	0
3056	Control-Fungi	NO	0
3066	Control-Fungi	NO	0.00666667
3077	Control-Fungi	NO	0

3093	Control-Fungi	NO	0
3011	Bacteria	NO	0.28333333
3018	Bacteria	YES	0.58333333
3035	Bacteria	YES	0.35
3042	Bacteria	YES	0.2
3050	Bacteria	YES	0.05666667
3072	Bacteria	YES	0.13333333
3081	Bacteria	YES	0.21666667
3086	Bacteria	YES	0.1
3012	Fungi	YES	0.19
3015	Fungi	YES	0.00666667
3036	Fungi	YES	0.2
3040	Fungi	YES	0.66666667
3052	Fungi	YES	0.66666667
3071	Fungi	YES	0.25
3084	Fungi	YES	0.42333333
3087	Fungi	YES	0.28333333
3007	Nematode	YES	0.1
3013	Nematode	YES	0
3031	Nematode	YES	0.15
3039	Nematode	YES	0.00666667
3051	Nematode	YES	0.18333333
3068	Nematode	YES	0.25666667
3082	Nematode	YES	0.03333333
3088	Nematode	YES	0.51666667
3008	Mix	YES	0.5
3016	Mix	YES	0.09
3033	Mix	YES	0.09
3041	Mix	YES	0.03333333
3053	Mix	YES	0.11666667
3070	Mix	YES	0.33333333
3079	Mix	YES	0.05
3089	Mix	YES	0.25
3010	Control	YES	0.91666667
3014	Control	YES	0
3034	Control	YES	0.7
3038	Control	YES	0.09
3049	Control	YES	0.33333333
3067	Control	YES	0.45
3080	Control	YES	0.20666667
3085	Control	YES	0.36666667
3009	Control-Fungi	YES	0.16666667
3017	Control-Fungi	YES	0.5
3032	Control-Fungi	YES	0.25
3037	Control-Fungi	YES	0.17333333
3054	Control-Fungi	YES	0.58333333
3069	Control-Fungi	YES	0.5
3083	Control-Fungi	YES	0.16666667
3090	Control-Fungi	YES	0.18333333

Larval recovery 2016

Plot	Beneficial	WCR	Larval recovery (mean nb)
3005	Bacteria	NO	0
3022	Bacteria	NO	0
3028	Bacteria	NO	0
3047	Bacteria	NO	1
3059	Bacteria	NO	0.5
3063	Bacteria	NO	0
3076	Bacteria	NO	0
3092	Bacteria	NO	0.5
3004	Fungi	NO	0
3019	Fungi	NO	0
3029	Fungi	NO	0
3045	Fungi	NO	0
3060	Fungi	NO	0.5
3065	Fungi	NO	0
3075	Fungi	NO	0
3091	Fungi	NO	0
3003	Nematode	NO	0
3023	Nematode	NO	0.5
3027	Nematode	NO	0
3048	Nematode	NO	0.5
3057	Nematode	NO	0
3062	Nematode	NO	0
3078	Nematode	NO	0
3096	Nematode	NO	0
3002	Mix	NO	0
3024	Mix	NO	0
3030	Mix	NO	0
3046	Mix	NO	0
3055	Mix	NO	0.5
3061	Mix	NO	0
3074	Mix	NO	0
3095	Mix	NO	0
3001	Control	NO	0.5
3020	Control	NO	0
3025	Control	NO	0
3044	Control	NO	0
3058	Control	NO	1.5
3064	Control	NO	0
3073	Control	NO	0
3094	Control	NO	0
3006	Control-Fungi	NO	0
3021	Control-Fungi	NO	0
3026	Control-Fungi	NO	0
3043	Control-Fungi	NO	0
3056	Control-Fungi	NO	0
3066	Control-Fungi	NO	0
3077	Control-Fungi	NO	0

3093	Control-Fungi	NO	0
3011	Bacteria	YES	2.5
3018	Bacteria	YES	0.5
3035	Bacteria	YES	1.5
3042	Bacteria	YES	0.5
3050	Bacteria	YES	3
3072	Bacteria	YES	2
3081	Bacteria	YES	1
3086	Bacteria	YES	1.5
3012	Fungi	YES	6.5
3015	Fungi	YES	4.5
3036	Fungi	YES	0.5
3040	Fungi	YES	2.5
3052	Fungi	YES	3
3071	Fungi	YES	2
3084	Fungi	YES	3
3087	Fungi	YES	5
3007	Nematode	YES	4
3013	Nematode	YES	0.5
3031	Nematode	YES	4
3039	Nematode	YES	2
3051	Nematode	YES	5
3068	Nematode	YES	0
3082	Nematode	YES	0.5
3088	Nematode	YES	6.5
3008	Mix	YES	6
3016	Mix	YES	2
3033	Mix	YES	3.5
3041	Mix	YES	1.5
3053	Mix	YES	2
3070	Mix	YES	4.5
3079	Mix	YES	1.5
3089	Mix	YES	4
3010	Control	YES	7
3014	Control	YES	1.5
3034	Control	YES	4.5
3038	Control	YES	1.5
3049	Control	YES	3.5
3067	Control	YES	5
3080	Control	YES	6
3085	Control	YES	5.5
3009	Control-Fungi	YES	2.5
3017	Control-Fungi	YES	6.5
3032	Control-Fungi	YES	9
3037	Control-Fungi	YES	1.5
3054	Control-Fungi	YES	8.5
3069	Control-Fungi	YES	3.5
3083	Control-Fungi	YES	0.5
3090	Control-Fungi	YES	5.5

Larval weight 2016

Plot	Beneficial	WCR	larval weight (mg)
3005	Bacteria	NO	.
3022	Bacteria	NO	.
3028	Bacteria	NO	.
3047	Bacteria	NO	0.234
3059	Bacteria	NO	.
3063	Bacteria	NO	.
3076	Bacteria	NO	.
3092	Bacteria	NO	0.247
3004	Fungi	NO	.
3019	Fungi	NO	.
3029	Fungi	NO	.
3045	Fungi	NO	.
3060	Fungi	NO	0.342
3065	Fungi	NO	.
3075	Fungi	NO	.
3091	Fungi	NO	.
3003	Nematode	NO	.
3023	Nematode	NO	0.106
3027	Nematode	NO	.
3048	Nematode	NO	0.217
3057	Nematode	NO	.
3062	Nematode	NO	.
3078	Nematode	NO	.
3096	Nematode	NO	.
3002	Mix	NO	.
3024	Mix	NO	.
3030	Mix	NO	.
3046	Mix	NO	.
3055	Mix	NO	0.092
3061	Mix	NO	.
3074	Mix	NO	.
3095	Mix	NO	.
3001	Control	NO	0.241
3020	Control	NO	.
3025	Control	NO	.
3044	Control	NO	.
3058	Control	NO	0.552
3064	Control	NO	.
3073	Control	NO	.
3094	Control	NO	.
3006	Control-Fungi	NO	.
3021	Control-Fungi	NO	.
3026	Control-Fungi	NO	.
3043	Control-Fungi	NO	.
3056	Control-Fungi	NO	.
3066	Control-Fungi	NO	.
3077	Control-Fungi	NO	.

3093	Control-Fungi	NO	.
3011	Bacteria	YES	0.482
3018	Bacteria	YES	0.231
3035	Bacteria	YES	0.2275
3042	Bacteria	YES	0.055
3050	Bacteria	YES	0.09
3072	Bacteria	YES	0.314
3081	Bacteria	YES	0.268
3086	Bacteria	YES	0.276
3012	Fungi	YES	0.769
3015	Fungi	YES	0.572
3036	Fungi	YES	0.18
3040	Fungi	YES	0.3465
3052	Fungi	YES	0.759
3071	Fungi	YES	0.266
3084	Fungi	YES	0.307
3087	Fungi	YES	0.6565
3007	Nematode	YES	0.6145
3013	Nematode	YES	0.123
3031	Nematode	YES	0.376
3039	Nematode	YES	0.925
3051	Nematode	YES	0.4655
3068	Nematode	YES	.
3082	Nematode	YES	0.187
3088	Nematode	YES	1.1145
3008	Mix	YES	0.757
3016	Mix	YES	0.2375
3033	Mix	YES	0.3925
3041	Mix	YES	0.201
3053	Mix	YES	0.2405
3070	Mix	YES	0.8075
3079	Mix	YES	0.2625
3089	Mix	YES	0.8045
3010	Control	YES	1.985
3014	Control	YES	0.1705
3034	Control	YES	0.665
3038	Control	YES	0.2185
3049	Control	YES	0.447
3067	Control	YES	0.6805
3080	Control	YES	1.696
3085	Control	YES	0.984
3009	Control-Fungi	YES	0.989
3017	Control-Fungi	YES	0.915
3032	Control-Fungi	YES	1.1335
3037	Control-Fungi	YES	0.345
3054	Control-Fungi	YES	1.0185
3069	Control-Fungi	YES	0.689
3083	Control-Fungi	YES	0.196
3090	Control-Fungi	YES	1.1115

Yield 2016

Plot	Beneficial	WCR	Weight (g)
3099	Bacteria	NO	1167.4
3117	Bacteria	NO	812.1
3127	Bacteria	NO	904.3
3134	Bacteria	NO	717.3
3150	Bacteria	NO	537.4
3157	Bacteria	NO	607.3
3180	Bacteria	NO	832.1
3184	Bacteria	NO	752.3
3100	Fungi	NO	1054.9
3119	Fungi	NO	886.8
3129	Fungi	NO	822.7
3137	Fungi	NO	915.4
3146	Fungi	NO	663.6
3158	Fungi	NO	745
3175	Fungi	NO	741.7
3185	Fungi	NO	604.9
3097	Nematode	NO	1013
3120	Nematode	NO	577.3
3131	Nematode	NO	855.6
3136	Nematode	NO	831.1
3145	Nematode	NO	700.3
3162	Nematode	NO	642
3179	Nematode	NO	868.7
3183	Nematode	NO	601.5
3101	Mix	NO	1120.5
3116	Mix	NO	850.1
3130	Mix	NO	727.6
3135	Mix	NO	856.8
3149	Mix	NO	602.5
3159	Mix	NO	700.3
3177	Mix	NO	1024.4
3186	Mix	NO	870.6
3098	Control	NO	1031.8
3115	Control	NO	1194
3128	Control	NO	627.6
3133	Control	NO	853.9
3147	Control	NO	605
3161	Control	NO	646.3
3178	Control	NO	817
3181	Control	NO	1156.5
3102	Control-Fungi	NO	1164.8
3118	Control-Fungi	NO	909
3132	Control-Fungi	NO	713.1
3138	Control-Fungi	NO	664.9
3148	Control-Fungi	NO	896.7
3160	Control-Fungi	NO	505.9
3176	Control-Fungi	NO	833.3

3182	Control-Fungi	NO	1053.5
3103	Bacteria	YES	1131
3114	Bacteria	YES	882.4
3123	Bacteria	YES	672.9
3143	Bacteria	YES	955.5
3151	Bacteria	YES	758.5
3166	Bacteria	YES	670.2
3170	Bacteria	YES	982
3187	Bacteria	YES	941.6
3105	Fungi	YES	1044.6
3109	Fungi	YES	1004.2
3122	Fungi	YES	748.5
3144	Fungi	YES	791.4
3153	Fungi	YES	764.7
3163	Fungi	YES	638.6
3174	Fungi	YES	992
3190	Fungi	YES	567
3107	Nematode	YES	899.2
3112	Nematode	YES	983.6
3124	Nematode	YES	742.9
3139	Nematode	YES	1035.3
3155	Nematode	YES	764.3
3167	Nematode	YES	503.7
3173	Nematode	YES	896.7
3189	Nematode	YES	690.1
3104	Mix	YES	1062.2
3111	Mix	YES	804
3121	Mix	YES	945.7
3141	Mix	YES	807
3154	Mix	YES	761.6
3164	Mix	YES	750.8
3169	Mix	YES	954.4
3188	Mix	YES	1004.1
3106	Control	YES	613.1
3113	Control	YES	820.9
3125	Control	YES	641.2
3142	Control	YES	874.6
3152	Control	YES	726.6
3165	Control	YES	776.6
3171	Control	YES	1000.9
3192	Control	YES	676.5
3108	Control-Fungi	YES	928.1
3110	Control-Fungi	YES	1003.8
3126	Control-Fungi	YES	600.8
3140	Control-Fungi	YES	1072.8
3156	Control-Fungi	YES	615.4
3168	Control-Fungi	YES	575.9
3172	Control-Fungi	YES	1016.6
3191	Control-Fungi	YES	505.2

Field design 2017

Plant damage/larval recovery plots

Block	Plot	Treatment	WCR		Beneficial	Code
1	1001	**12**	add	1	Control-Fungi	**C-F***
1	1002	**8**	add	2	Fungi	**F***
1	1003	**11**	add	3	Control	**C***
1	1004	**9**	add	4	Nematode	**N***
1	1005	**7**	add	5	Bacteria	**B***
1	1006	**10**	add	6	Mix	**MIX***
1	1007	**4**	neg	1	Mix	**MIX**
1	1008	**6**	neg	2	Control-Fungi	**C-F**
1	1009	**2**	neg	3	Fungi	**F**
1	1010	**1**	neg	4	Bacteria	**B**
1	1011	**3**	neg	5	Nematode	**N**
1	1012	**5**	neg	6	Control	**C**
2	1013	**9**	add	1	Nematode	**N***
2	1014	**8**	add	2	Fungi	**F***
2	1015	**12**	add	3	Control-Fungi	**C-F***
2	1016	**11**	add	4	Control	**C***
2	1017	**10**	add	5	Mix	**MIX***
2	1018	**7**	add	6	Bacteria	**B***
2	1019	**5**	neg	1	Control	**C**
2	1020	**3**	neg	2	Nematode	**N**
2	1021	**1**	neg	3	Bacteria	**B**
2	1022	**4**	neg	4	Mix	**MIX**
2	1023	**2**	neg	5	Fungi	**F**
2	1024	**6**	neg	6	Control-Fungi	**C-F**
3	1025	**4**	neg	1	Mix	**MIX**
3	1026	**1**	neg	2	Bacteria	**B**
3	1027	**5**	neg	3	Control	**C**
3	1028	**3**	neg	4	Nematode	**N**
3	1029	**2**	neg	5	Fungi	**F**
3	1030	**6**	neg	6	Control-Fungi	**C-F**
3	1031	**8**	add	1	Fungi	**F***
3	1032	**9**	add	2	Nematode	**N***
3	1033	**7**	add	3	Bacteria	**B***
3	1034	**11**	add	4	Control	**C***
3	1035	**10**	add	5	Mix	**MIX***
3	1036	**12**	add	6	Control-Fungi	**C-F***
4	1037	**8**	add	1	Fungi	**F***
4	1038	**9**	add	2	Nematode	**N***
4	1039	**12**	add	3	Control-Fungi	**C-F***
4	1040	**11**	add	4	Control	**C***
4	1041	**10**	add	5	Mix	**MIX***
4	1042	**7**	add	6	Bacteria	**B***
4	1043	**5**	neg	1	Control	**C**
4	1044	**2**	neg	2	Fungi	**F**
4	1045	**1**	neg	3	Bacteria	**B**
4	1046	**6**	neg	4	Control-Fungi	**C-F**

4	1047	4	neg	5	Mix	MIX
4	1048	3	neg	6	Nematode	N
5	1049	12	add	1	Control-Fungi	C-F*
5	1050	10	add	2	Mix	MIX*
5	1051	8	add	3	Fungi	F*
5	1052	11	add	4	Control	C*
5	1053	9	add	5	Nematode	N*
5	1054	7	add	6	Bacteria	B*
5	1055	1	neg	1	Bacteria	B
5	1056	2	neg	2	Fungi	F
5	1057	4	neg	3	Mix	MIX
5	1058	6	neg	4	Control-Fungi	C-F
5	1059	3	neg	5	Nematode	N
5	1060	5	neg	6	Control	C
6	1061	9	add	1	Nematode	N*
6	1062	11	add	2	Control	C*
6	1063	8	add	3	Fungi	F*
6	1064	10	add	4	Mix	MIX*
6	1065	7	add	5	Bacteria	B*
6	1066	12	add	6	Control-Fungi	C-F*
6	1067	6	neg	1	Control-Fungi	C-F
6	1068	5	neg	2	Control	C
6	1069	1	neg	3	Bacteria	B
6	1070	3	neg	4	Nematode	N
6	1071	4	neg	5	Mix	MIX
6	1072	2	neg	6	Fungi	F
7	1073	2	neg	1	Fungi	F
7	1074	4	neg	2	Mix	MIX
7	1075	6	neg	3	Control-Fungi	C-F
7	1076	3	neg	4	Nematode	N
7	1077	1	neg	5	Bacteria	B
7	1078	5	neg	6	Control	C
7	1079	11	add	1	Control	C*
7	1080	7	add	2	Bacteria	B*
7	1081	12	add	3	Control-Fungi	C-F*
7	1082	10	add	4	Mix	MIX*
7	1083	8	add	5	Fungi	F*
7	1084	9	add	6	Nematode	N*
8	1085	2	neg	1	Fungi	F
8	1086	5	neg	2	Control	C
8	1087	6	neg	3	Control-Fungi	C-F
8	1088	4	neg	4	Mix	MIX
8	1089	3	neg	5	Nematode	N
8	1090	1	neg	6	Bacteria	B
8	1091	7	add	1	Bacteria	B*
8	1092	8	add	2	Fungi	F*
8	1093	9	add	3	Nematode	N*
8	1094	10	add	4	Mix	MIX*
8	1095	11	add	5	Control	C*
8	1096	12	add	6	Control-Fungi	C-F*

Root damage 2017

Plot	WCR	Beneficial	Damage (1-3)
1005	add	Bacteria	0.766666667
1018	add	Bacteria	0.7
1033	add	Bacteria	1.166666667
1042	add	Bacteria	0.166666667
1054	add	Bacteria	1
1065	add	Bacteria	1.5
1080	add	Bacteria	0.116666667
1091	add	Bacteria	0.933333333
1010	neg	Bacteria	0
1021	neg	Bacteria	0
1026	neg	Bacteria	0.003333333
1045	neg	Bacteria	0.005
1055	neg	Bacteria	0
1069	neg	Bacteria	0.003333333
1077	neg	Bacteria	0
1090	neg	Bacteria	0.006666667
1003	add	Control	1.35
1016	add	Control	0.916666667
1034	add	Control	1.333333333
1040	add	Control	0.216666667
1052	add	Control	2.083333333
1062	add	Control	1.083333333
1079	add	Control	0.75
1095	add	Control	1.666666667
1012	neg	Control	0
1019	neg	Control	0
1027	neg	Control	0.003333333
1043	neg	Control	0
1060	neg	Control	0
1068	neg	Control	0
1078	neg	Control	0
1086	neg	Control	0
1001	add	Control-Fungi	0.253333333
1015	add	Control-Fungi	2.333333333
1036	add	Control-Fungi	1.166666667
1039	add	Control-Fungi	2.083333333
1049	add	Control-Fungi	2
1066	add	Control-Fungi	1.25
1081	add	Control-Fungi	1.666666667
1096	add	Control-Fungi	1.916666667
1008	neg	Control-Fungi	0
1024	neg	Control-Fungi	0.003333333
1030	neg	Control-Fungi	0
1046	neg	Control-Fungi	0.003333333
1058	neg	Control-Fungi	0.016666667
1067	neg	Control-Fungi	0.016666667
1075	neg	Control-Fungi	0

1087	neg	Control-Fungi	0.016666667
1002	add	Fungi	1.083333333
1014	add	Fungi	3
1031	add	Fungi	1.75
1037	add	Fungi	2.416666667
1051	add	Fungi	0.683333333
1063	add	Fungi	2.75
1083	add	Fungi	0.866666667
1092	add	Fungi	1.916666667
1009	neg	Fungi	0.006666667
1023	neg	Fungi	0
1029	neg	Fungi	0.006666667
1044	neg	Fungi	0.003333333
1056	neg	Fungi	0.03
1072	neg	Fungi	0.003333333
1073	neg	Fungi	0
1085	neg	Fungi	0.03333333
1006	add	Mix	0.25
1017	add	Mix	0.95
1035	add	Mix	2
1041	add	Mix	0.7
1050	add	Mix	0.683333333
1064	add	Mix	1
1082	add	Mix	0.833333333
1094	add	Mix	0.833333333
1007	neg	Mix	0.003333333
1022	neg	Mix	0
1025	neg	Mix	0.173333333
1047	neg	Mix	0
1057	neg	Mix	0
1071	neg	Mix	0
1074	neg	Mix	0
1088	neg	Mix	0
1004	add	Nematode	1.1
1013	add	Nematode	0.283333333
1032	add	Nematode	1.5
1038	add	Nematode	1.083333333
1053	add	Nematode	1.833333333
1061	add	Nematode	1.5
1084	add	Nematode	0.333333333
1093	add	Nematode	0.333333333
1011	neg	Nematode	0
1020	neg	Nematode	0
1028	neg	Nematode	0
1048	neg	Nematode	0.003333333
1059	neg	Nematode	0
1070	neg	Nematode	0
1076	neg	Nematode	0
1089	neg	Nematode	0

Larval recovery 2017

Plot	WCR	Beneficial	Larval recovery (mean nb)
1010	NO	Bacteria	0
1021	NO	Bacteria	0
1026	NO	Bacteria	0
1045	NO	Bacteria	0
1055	NO	Bacteria	0
1069	NO	Bacteria	0.5
1077	NO	Bacteria	0
1090	NO	Bacteria	1
1009	NO	Fungi	0
1023	NO	Fungi	0
1029	NO	Fungi	1
1044	NO	Fungi	0
1056	NO	Fungi	0
1072	NO	Fungi	0
1073	NO	Fungi	0
1085	NO	Fungi	0
1011	NO	Nematode	0
1020	NO	Nematode	0
1028	NO	Nematode	0
1048	NO	Nematode	0
1059	NO	Nematode	0
1070	NO	Nematode	1
1076	NO	Nematode	0.5
1089	NO	Nematode	0
1007	NO	Mix	0
1022	NO	Mix	0
1025	NO	Mix	0
1047	NO	Mix	0
1057	NO	Mix	0
1071	NO	Mix	0
1074	NO	Mix	0
1088	NO	Mix	0
1012	NO	Control	0
1019	NO	Control	0
1027	NO	Control	0
1043	NO	Control	1
1060	NO	Control	0.5
1068	NO	Control	0
1078	NO	Control	0
1086	NO	Control	0
1008	NO	Control-Fungi	0
1024	NO	Control-Fungi	0
1030	NO	Control-Fungi	0
1046	NO	Control-Fungi	0
1058	NO	Control-Fungi	0
1067	NO	Control-Fungi	0
1075	NO	Control-Fungi	0

1087	NO	Control-Fungi	0
1005	YES	Bacteria	4
1018	YES	Bacteria	4
1033	YES	Bacteria	1.5
1042	YES	Bacteria	2
1054	YES	Bacteria	3
1065	YES	Bacteria	5.5
1080	YES	Bacteria	7.5
1091	YES	Bacteria	0.5
1002	YES	Fungi	1.5
1014	YES	Fungi	6.5
1031	YES	Fungi	7.5
1037	YES	Fungi	21.5
1051	YES	Fungi	3
1063	YES	Fungi	4.5
1083	YES	Fungi	6
1092	YES	Fungi	5.5
1004	YES	Nematode	6.5
1013	YES	Nematode	3.5
1032	YES	Nematode	3.5
1038	YES	Nematode	5
1053	YES	Nematode	2
1061	YES	Nematode	2.5
1084	YES	Nematode	1
1093	YES	Nematode	9
1006	YES	Mix	1.5
1017	YES	Mix	2
1035	YES	Mix	4.5
1041	YES	Mix	5
1050	YES	Mix	4.5
1064	YES	Mix	4.5
1082	YES	Mix	6
1094	YES	Mix	5
1003	YES	Control	4
1016	YES	Control	1.5
1034	YES	Control	3
1040	YES	Control	1
1052	YES	Control	1.5
1062	YES	Control	4
1079	YES	Control	4
1095	YES	Control	6
1001	YES	Control-Fungi	5
1015	YES	Control-Fungi	11.5
1036	YES	Control-Fungi	3
1039	YES	Control-Fungi	4.5
1049	YES	Control-Fungi	2.5
1066	YES	Control-Fungi	3.5
1081	YES	Control-Fungi	3.5
1096	YES	Control-Fungi	10

Larval weight 2017

Plot	WCR	Beneficial	larval weight (mg)
1005	YES	Bacteria	1.2575
1018	YES	Bacteria	1.02
1033	YES	Bacteria	0.78333333
1042	YES	Bacteria	1.095
1054	YES	Bacteria	0.85666667
1065	YES	Bacteria	1.26454545
1080	YES	Bacteria	1.02466667
1091	YES	Bacteria	.
1002	YES	Fungi	0.84
1014	YES	Fungi	0.95307692
1031	YES	Fungi	0.98733333
1037	YES	Fungi	0.93348837
1051	YES	Fungi	0.81666667
1063	YES	Fungi	1.20333333
1083	YES	Fungi	1.38916667
1092	YES	Fungi	1.34909091
1004	YES	Nematode	1.04307692
1013	YES	Nematode	1.34285714
1032	YES	Nematode	0.80428571
1038	YES	Nematode	1.01
1053	YES	Nematode	0.935
1061	YES	Nematode	1.228
1084	YES	Nematode	1.005
1093	YES	Nematode	0.56388889
1006	YES	Mix	1.26666667
1017	YES	Mix	2.0025
1035	YES	Mix	0.94777778
1041	YES	Mix	1.39
1050	YES	Mix	1.29444444
1064	YES	Mix	1.08222222
1082	YES	Mix	1.47166667
1094	YES	Mix	1.414
1003	YES	Control	1.1825
1016	YES	Control	1.63333333
1034	YES	Control	0.75333333
1040	YES	Control	0.64
1052	YES	Control	1.08666667
1062	YES	Control	1.3825
1079	YES	Control	0.5075
1095	YES	Control	1.09
1001	YES	Control-Fungi	1.554
1015	YES	Control-Fungi	1.13565217
1036	YES	Control-Fungi	0.49333333
1039	YES	Control-Fungi	0.88222222
1049	YES	Control-Fungi	1.12
1066	YES	Control-Fungi	1.40857143
1081	YES	Control-Fungi	0.64428571

1096	YES	Control-Fungi	0.8685
1010	NO	Bacteria	.
1021	NO	Bacteria	.
1026	NO	Bacteria	.
1045	NO	Bacteria	.
1055	NO	Bacteria	.
1069	NO	Bacteria	0.06
1077	NO	Bacteria	.
1090	NO	Bacteria	1.215
1009	NO	Fungi	.
1023	NO	Fungi	.
1029	NO	Fungi	0.47
1044	NO	Fungi	.
1056	NO	Fungi	.
1072	NO	Fungi	.
1073	NO	Fungi	.
1085	NO	Fungi	.
1011	NO	Nematode	.
1020	NO	Nematode	.
1028	NO	Nematode	.
1048	NO	Nematode	.
1059	NO	Nematode	.
1070	NO	Nematode	0.065
1076	NO	Nematode	1.6
1089	NO	Nematode	.
1007	NO	Mix	.
1022	NO	Mix	.
1025	NO	Mix	.
1047	NO	Mix	.
1057	NO	Mix	.
1071	NO	Mix	.
1074	NO	Mix	.
1088	NO	Mix	.
1012	NO	Control	.
1019	NO	Control	.
1027	NO	Control	.
1043	NO	Control	2.17
1060	NO	Control	1.78
1068	NO	Control	.
1078	NO	Control	.
1086	NO	Control	.
1008	NO	Control-Fungi	.
1024	NO	Control-Fungi	.
1030	NO	Control-Fungi	.
1046	NO	Control-Fungi	.
1058	NO	Control-Fungi	.
1067	NO	Control-Fungi	.
1075	NO	Control-Fungi	.
1087	NO	Control-Fungi	.

Yield 2017

Plot	WCR	Beneficial	Weight (g)
1102	NO	Bacteria	443.783688
1110	NO	Bacteria	1227.53008
1128	NO	Bacteria	755.98227
1138	NO	Bacteria	740.594697
1148	NO	Bacteria	816.292
1164	NO	Bacteria	936.902344
1180	NO	Bacteria	305.454887
1186	NO	Bacteria	841.72695
1101	NO	Fungi	725.445255
1109	NO	Fungi	835.875
1130	NO	Fungi	1190.05556
1133	NO	Fungi	726.046763
1146	NO	Fungi	587.652174
1167	NO	Fungi	1056.24809
1177	NO	Fungi	1112.24609
1185	NO	Fungi	712.369919
1098	NO	Nematode	1008.368
1112	NO	Nematode	568.036885
1131	NO	Nematode	544.665441
1135	NO	Nematode	740.582677
1147	NO	Nematode	572.934307
1168	NO	Nematode	1147.24409
1176	NO	Nematode	976.150376
1181	NO	Nematode	1282.45652
1100	NO	Mix	824.272358
1111	NO	Mix	897.25188
1132	NO	Mix	429.951493
1134	NO	Mix	829.46831
1150	NO	Mix	907.556
1163	NO	Mix	991.03125
1179	NO	Mix	794.267361
1183	NO	Mix	1017.172
1097	NO	Control	1290.43852
1114	NO	Control	771.776
1127	NO	Control	838.357664
1137	NO	Control	888.666667
1149	NO	Control	587.304688
1165	NO	Control	505.92
1178	NO	Control	1196.38372
1184	NO	Control	677.651079
1099	NO	Control-Fungi	664.30315
1113	NO	Control-Fungi	682.834615
1129	NO	Control-Fungi	970.164234
1136	NO	Control-Fungi	626.488372
1145	NO	Control-Fungi	1197.23358
1166	NO	Control-Fungi	1101.78276
1175	NO	Control-Fungi	727.190141

1182	NO	Control-Fungi	1117.14815
1108	YES	Bacteria	999.566929
1118	YES	Bacteria	924.639098
1125	YES	Bacteria	1053.44643
1139	YES	Bacteria	948.023256
1155	YES	Bacteria	684.165441
1161	YES	Bacteria	655.822222
1171	YES	Bacteria	1007.748
1190	YES	Bacteria	988.620301
1107	YES	Fungi	842.698529
1115	YES	Fungi	688.774074
1124	YES	Fungi	665.503571
1144	YES	Fungi	639.79927
1152	YES	Fungi	658.467153
1159	YES	Fungi	666.172535
1173	YES	Fungi	1009.66783
1192	YES	Fungi	1322.956
1105	YES	Nematode	1179.48162
1119	YES	Nematode	990.277778
1122	YES	Nematode	924.985294
1142	YES	Nematode	1209.58271
1153	YES	Nematode	496.120155
1157	YES	Nematode	290.914179
1169	YES	Nematode	1316.91729
1189	YES	Nematode	1117.612
1106	YES	Mix	1190.32677
1117	YES	Mix	1183.32813
1123	YES	Mix	928.189781
1143	YES	Mix	313.640152
1151	YES	Mix	493.58156
1160	YES	Mix	907.433824
1174	YES	Mix	147.306569
1191	YES	Mix	837.446043
1103	YES	Control	1072.82963
1116	YES	Control	551.406716
1126	YES	Control	874.507634
1140	YES	Control	797.865248
1156	YES	Control	889.043796
1162	YES	Control	1055.59559
1172	YES	Control	913.995935
1188	YES	Control	1158.53759
1104	YES	Control-Fungi	494.518382
1120	YES	Control-Fungi	1026.19466
1121	YES	Control-Fungi	610.240741
1141	YES	Control-Fungi	900.419847
1154	YES	Control-Fungi	905.753571
1158	YES	Control-Fungi	479.869919
1170	YES	Control-Fungi	770.434109
